# Phase-II monitoring of the lower truncated proportional hazard rate process based on progressive Type-II censoring

**DOI:** 10.1371/journal.pone.0322996

**Published:** 2025-06-03

**Authors:** Shohreh Enami, Osama Abdulaziz Alamri

**Affiliations:** 1 Department of Statistics, Payame Noor University, Tehran, Iran; 2 Department of Statistics, University of Tabuk, Tabuk, Saudi Arabia; University of Hamburg: Universitat Hamburg, GERMANY

## Abstract

The problem of monitoring statistical processes using complete data has been extensively studied by researchers. However, in fields such as reliability engineering and lifetime experiments, complete samples are often not available. To address this gap, we introduce four control charts designed for monitoring both parameters of a family of distributions known as the lower truncated proportional hazard rate model, specifically under progressively Type-II censoring. Three of these control charts are exponentially weighted moving average (EWMA) charts that utilize the likelihood ratio statistic and maximum likelihood estimators. The fourth chart is based on a novel weighted log-likelihood ratio statistic. We conduct a Monte Carlo simulation study to assess the performance of the proposed control charts. Finally, we present a practical example to illustrate the application of our methods.

## Introduction

Evaluating the lifetime of a product is a critical aspect of product reliability. Research on product lifetime has been conducted across various fields, including industrial manufacturing, medicine, and reliability engineering.

To make inferences about a product’s lifetime under standard manufacturing conditions and to swiftly identify any defective items, it is essential to conduct life tests and reliability experiments. Statistical Process Monitoring (SPM) serves as a widely used tool for making inferences regarding process performance and has been implemented extensively in industrial settings. Within SPM, control charts play a pivotal role as graphical tools for monitoring process status and determining whether the process is in control (IC). The development of efficient control charts is crucial for the continuous monitoring of product lifetime, enabling real-time alerts when signs of deterioration are detected.

The EWMA control chart was first introduced by [1]. It was designed to detect small shifts that allows for quicker detection of changes in the process, making it a valuable tool in quality control and monitoring. Due to ability of this control chart, many researchers have extended to several models in the recent years. For instance, see [2–4].

In reliability engineering and life testing, we usually do not observe complete data and we come across the censored data to save time and cost involving the experiment. Some researchers focused on developing control charts based on censored data. For instance, [5] proposed an EWMA control chart to monitor the Weibull mean based on random censored data. [6] proposed a control chart to monitor the shape parameter of a Weibull process under Type-II censoring. [7] introduced an EMWA control chart for the Poisson exponential lifetime model under Type-I censored data. [8] proposed a cumulative sum (CUSUM) control chart to monitor the mean of the Weibull lifetime model with the fixed shape parameter. [9] discussed a weighted likelihood control chart based on the Weibull distributed observations under random censoring. [10] proposed a weighted EWMA chart for Weibull distribution in Type-I censored samples. [11] proposed some control charts for monitoring the lower Weibull percentiles under Type-II censoring. [12, 13] proposed some control charts under Type-II censored reliability tests by assuming that the lifetimes follow the Weibull distribution with fixed and stable shape parameter.

Unlike conventional censoring, progressive censoring offers more flexibility by permitting the removal of certain living subjects during the experiment. Progressive Type-II censoring has been introduced by [14, 15]. In recent years, a lot of researches has been made based on progressively Type-II censored samples; for comprehensive discussions in the theory of this topic, one may refer to [16, 17]. In progressive Type-II censoring, it is assumed that the removals of still operating units are carried out at observed failure times and that the censoring scheme $r=(r_{1},r_{2},\ldots ,r_{m})$ is known in advance. Moreover, the number of units (*n*) and the number of observed failure times (*m*) are prefixed. Starting all *n* units at the same time, the first progressive censoring step takes place at the observation of the first failure time *X*_1:*m*:*n*_, at this time, *r*_1_ units are randomly chosen from the still operating units and withdrawn from the experiment. Then, the experiment continues with the reduced sample size $n\;-\;r_{1}\;-\;1$. After observing the next failure at time *X*_2:*m*:*n*_, *r*_2_ units are randomly removed from $n\;-\;r_{1}\;-\;2$ active units. This process continues until the *m*th failure is observed. Then, the experiment ends, and $X_{1:m:n}\leq \cdots \leq X_{m:m:n}$ are said to be progressively Type-II censored order statistics. This scheme includes as special cases the complete sample situation (when *m* = *n* and $r_{1}=\cdots =r_{m}=0$) and the conventional Type-II right censoring situation (when $r_{1}=\cdots =r_{m\;-\;1}=0$ and $r_{m}=n\;-\;m$). The conventional Type-II right censoring scheme arises in a life-testing experiment whenever the experimenter can not observe the objects with the high lifetimes and one is forced to only record the first *m* smallest lifetimes of *n* objects for inference. But in the setting of the progressive Type-II censoring, the experimenter may choose different censoring schemes $r=(r_{1},r_{2},\ldots ,r_{m})$ with fixed *m* and *n* to get the different inferences. This property of progressive Type-II censoring allows the experimenter to choose a censoring scheme to get the optimal inference. This subject provides valuable design ideas to reliability practitioners and evidence for the usefulness and efficiency of progressive Type-II right censoring as compared to conventional Type-II right censoring. For more details, we refer to [16].

Recently, developing control charts based on progressively Type-II censored samples has been considered by some researchers. For instance, [18] proposed a control chart to monitor the shape parameter of the Weibull distributed process under progressive Type-II censoring. [19] introduced some control charts for two-parameter exponential distribution for monitoring the location and scale parameters, simultaneously. [20] proposed some control charts for monitoring the scale parameter and also joint shape and scale parameters in the Weibull distributed process.

Modern products typically exhibit high reliability, and models that include small values in their domain often fail to accurately describe the lifetimes of such products. To address this issue, [21] introduced the lower truncated proportional hazard rate (LTPHR) model. The proportional hazard rate (PHR) model is a significant framework in reliability theory and various other fields. A random variable is said to follow the PHR model, if its survival function can be expressed as ${\bar{F}}^{\lambda }(x)$ where $\bar{F}(x)$ is a baseline absolutely continuous survival function and λ>0. For a more comprehensive discussion about the PHR model, we refer to [22–24]. A prevalent way to describe a random variable having a certain lower endpoint is a lower truncation of the well-known distributions. Let $\bar{F}(x)$, x∈ℝ be the survival function of a random variable *X*. The lower truncated version of *X* at point μ is defined as X|X≥μ with the corresponding survival function $\bar{F}(x)/\bar{F}(\mu )$, x≥μ. By this idea, a random variable *X* is said to follow the lower truncated proportional hazard rate model if its survival function can be expressed as $\bar{G}(x;\mu ,\lambda )={\left[\bar{F}(x)/\bar{F}(\mu )\right]}^{\lambda }$, x≥μ>0, λ>0, where $\bar{F}(x)$ is a baseline absolutely continuous survival function with the corresponding density function *f*(*x*). In this setting, we denote $X\sim \text{LTPHR}(\bar{F}(x),\mu ,\lambda )$. The LTPHR model has been recently considered in some research. For instance, see [25, 26].

In this paper, we focus on designing control charts for Phase-II joint monitoring of the parameters μ and λ in a LTPHR process, utilizing progressively Type-II censored data. Sect 1 introduces four distinct control charts tailored for this purpose. We discuss about the implementation design in Sect 2. A comprehensive simulation study is presented in Sect 3, evaluating the performance of our proposed control charts under various scenarios. Sect 4 demonstrates the application of our methods through an analysis of a real dataset. In Sect 5 we make a conclusion and the R codes used in simulation study are provided in Sect 6.

## 1 Control charts

In this section, we present control charts designed to identify potential shifts in both parameters of the lower truncated proportional hazard rate model under a progressive Type-II censoring scheme.

In Phase II, the batch of progressive Type-II censored samples $\left\{X_{i},\;i=1,2,\ldots \right\}$ from a LTPHR model are collected sequentially over time with the same censoring scheme $r=(r_{1},\ldots ,r_{m})$. Let $\left(\mu_{0},\lambda_{0}\right)$ be the IC value of the parameter vector (μ,λ).

### 1.1 EWMA control chart based on the likelihood ratio

In this subsection, we provide an EWMA control chart based on the likelihood ratio (EWMA-LR) to monitor the LTPHR process of progressively Type-II censored data.

Let $X_{i}$ be the *i*th batch of progressively Type-II censored sample from $\text{LTPHR}(\bar{F}(x),\mu ,\lambda )$ with the observation $x_{i}=(x_{i1},\ldots ,x_{im})$. According to [16] the likelihood function corresponding to $X_{i}$ can be written as


$$L_{i}(\mu ,\lambda )=\left(\prod_{j=1}^{m}\eta_{j}f(x_{ij})\right){\left(\frac{\lambda }{\bar{F}(\mu )}\right)}^{m}\prod_{j=1}^{m}{\left[\frac{\bar{F}(x_{ij})}{\bar{F}(\mu )}\right]}^{\lambda (1+r_{j})-1}{\text{I}}_{\left[x_{i1}>\mu \right]},$$

with the corresponding log-likelihood

$$l_{i}(\mu ,\lambda )=\left[c+m\ln\lambda -m\ln\bar{F}(\mu )+\sum_{j=1}^{m}\left(\lambda (1+r_{j})-1\right)\ln\frac{\bar{F}(x_{ij})}{\bar{F}(\mu )}\right]{\text{I}}_{\left[x_{i1}>\mu \right]},$$ (1)

where *c* is a free of (μ,λ), $\eta_{j}=\sum_{k=j}^{m}(1+r_{k})$, and ${\text{I}}_{\left[.\right]}$ denotes the indicator function. The MLEs of μ and λ based on the *i*th batch sample, denoted by ${\hat{\mu }}_{i}$ and ${\hat{\lambda }}_{i}$ respectively, are obtained by maximizing the function *L*_*i*_. Based on Theorem 1 of [21], MLEs of the parameters are obtained as the following:

$${\hat{\mu }}_{i}=x_{i1},{\hat{\lambda }}_{i}=m{\left[-\sum_{j=1}^{m}(1+r_{j})\ln\frac{\bar{F}(x_{ij})}{\bar{F}({\hat{\mu }}_{i})}\right]}^{-1}.$$ (2)

Let $\left(\mu_{0},\lambda_{0}\right)$ be the IC value of (μ,λ). The monitoring statistic, denoted by *LR*_*i*_, based on the likelihood ratio, is given by

$$LR_{i}=\ln\frac{L_{i}({\hat{\mu }}_{i},{\hat{\lambda }}_{i})}{L_{i}(\mu_{0},\lambda_{0})}=\left\{ \begin{array}{c} \infty ,\;\;\;\;{\hat{\mu }}_{i}<\mu_{0},\\ m\left[\frac{\lambda_{0}}{{\hat{\lambda }}_{i}}-\ln\frac{\lambda_{0}}{{\hat{\lambda }}_{i}}-1\right]+n\lambda_{0}\ln\frac{\bar{F}(\mu_{0})}{\bar{F}({\hat{\mu }}_{i})},\;{\hat{\mu }}_{i}>\mu_{0}. \end{array}\right.$$ (3)

Therefore, the charting statistic of the EWMA-LR control chart is given by

$$EL_{i}=(1-\gamma )EL_{i-1}+\gamma LR_{i},\;i=1,2,\ldots ,$$ (4)

where γ is a smoothing parameter and $EL_{0}=\text{E}[LR_{i}]$. The smoothing parameter γ plays a crucial role in the EWMA control chart, as it determines the weight assigned to recent observations. This parameter directly influences the smoothness and sensitivity of the control chart in response to process changes. A higher value of γ gives more weight to recent observations, making the control chart more responsive to immediate changes in the process. While this heightened sensitivity can be beneficial for quick detection of shifts, it also increases the rate of false alarms. Conversely, a smaller value of γ places greater emphasis on past observations, resulting in a smoother control chart. This approach reduces sensitivity to immediate changes, which may decrease the rate of false alarms but can also delay the detection of small shifts in the process. In summary, selecting an appropriate value for γ is essential for balancing sensitivity and false alarm rates in EWMA control charts. In most practical applications, one may consider 0.05≤γ≤0.25.

Note that *EL*_0_ does not have a closed-form expression and can be computed using Monte Carlo simulations. The EWMA-LR control chart generates a signal when *EL*_*i*_>*h* , where the threshold value *h* is selected to achieve a specified expected IC average run length (${\text{ARL}}_{0}$).

Based on Theorem 2.1 of [25], $2m\lambda_{0}/{\hat{\lambda }}_{i}$ follows the chi-square distribution with 2*m*–2 degrees of freedom and $\bar{F}({\hat{\mu }}_{i})/\bar{F}(\mu_{0})$ follows the beta distribution with the parameter vector $\left(n\lambda_{0},1\right)$. Thus, for an IC process, the distribution of *LR*_*i*_ only depends on *n* and *m* and is free of the censoring scheme r. Hence, the EWMA-LR control chart for the LTPHR model under progressive Type-II censoring does not depend on the censoring scheme.

### 1.2 EWMA-Max-MLE control chart

Here we utilize the idea of [27] to construct EWMA-Max-MLE charting statistic. Based on Theorem 2.1 of [25], we have


$$Z_{1i}=\frac{\bar{F}({\hat{\mu }}_{i})}{\bar{F}(\mu_{0})}\sim \text{B}(n\lambda_{0},1),\;Z_{2i}=\frac{2m\lambda_{0}}{{\hat{\lambda }}_{i}}\sim \chi_{2m-2}^{2},\;Z_{1i}⟂Z_{2i},\;i=1,2,\ldots ,$$

where $\text{B}(n\lambda_{0},1)$ denotes the beta distribution with the parameter vector $\left(n\lambda_{0},1\right)$ and $\chi_{2m-2}^{2}$ denotes the chi-squared distribution with 2*m*–2 degrees of freedom.

By the probability integral transform, $T_{1i}=\Phi^{-1}\left(F_{\text{B}(n\lambda_{0},1)}(Z_{1i})\right)$ and $T_{2i}=\Phi^{-1}\left(F_{\chi_{2m-2}^{2}}(Z_{2i})\right)$ independently follow the standard normal distribution when the process is IC, where $F_{\text{B}(n\lambda_{0},1)}$ denotes the cumulative distribution function of the beta distribution with parameter $\left(n\lambda_{0},1\right)$, $F_{\chi_{2m-2}^{2}}$ denotes the cumulative distribution function of the chi-squared distribution with 2*m*–2 degrees of freedom, and $\Phi^{-1}$ denotes the quantile function of the standard normal distribution.

Define $S_{i}=\max(|T_{1i}|,|T_{2i}|)$. An EWMA-Max-MLE control charting statistic can be obtained as


$$EM_{i}=(1-\gamma )EM_{i-1}+\gamma S_{i},\;i=1,2,\ldots ,$$

where $EM_{0}=\text{E}[S_{i}]$ such that according to [19], we have *EM*_0_ = 1.128379. The EWMA-Max-MLE control chart generates signals when *EL*_*i*_>*h*, where the value of *h* is selected to attain a specified expected ${\text{ARL}}_{0}$.

It is clear that the distribution of *S*_*i*_ only depends on *n* and *m* and is free of the censoring scheme r. Hence, to construct the EWMA-Max-MLE control chart for the LTPHR model under progressive Type-II censoring, the censoring scheme is not required.

### 1.3 EWMA control chart based on the Manhattan distance

In this subsection, we provide an EWMA control chart based on the Manhattan distance (EWMA-MD) to monitor the LTPHR model process of progressively Type-II censored data. For this purpose, we use the Manhattan distance between the points $\left(-\lambda_{0}\ln\bar{F}({\hat{\mu }}_{i}),\lambda_{0}/{\hat{\lambda }}_{i}\right)$ and $\left(-\lambda_{0}\ln\bar{F}(\mu_{0}),1\right)$, of the form $MD_{i}=\lambda_{0}|\ln\frac{\bar{F}(\mu_{0})}{\bar{F}({\hat{\mu }}_{i})}|+|\frac{\lambda_{0}}{{\hat{\lambda }}_{i}}-1|$, in *i*th batch sample. The charting statistic of the EWMA-MD control chart is given by

$$EMD_{i}=(1-\gamma )EMD_{i-1}+\gamma MD_{i},\;i=1,2,\ldots ,$$ (5)

where γ is a smoothing parameter and $EMD_{0}=\text{E}[MD_{i}]$. The EWMA-MD control chart generates signals when *EMD*_*i*_>*h*, where the value of *h* is selected to get a specified expected ${\text{ARL}}_{0}$. Also, by a similar argument to the previous subsections, we conclude that the EWMA-MD control chart for the LTPHR model under progressive Type-II censoring does not depend on the censoring scheme and only depends on *m* and *n*.

### 1.4 WL control chart

Hypothesis testing based on the weighted log-likelihood was proposed by [28]. Then, [29] used this idea to design the control charts for monitoring the Poisson count process. Recently, [20] proposed a WL control chart for monitoring a Weibull process under Type-II censoring by the weighted log-likelihood function.

We present a WL chart statistic to achieve sequential monitoring. From time 1 to *t*, by Eq (1), sum of the weighted log-likelihood functions can be written as


$$wl_{t}(\mu ,\lambda )=\sum_{i=1}^{t}w_{i,\gamma }l_{i}(\mu ,\lambda )$$

$$=\sum_{i=1}^{t}w_{i,\gamma }\left[c+m\ln\lambda -m\ln\bar{F}(\mu )+\sum_{j=1}^{m}\left(\lambda (1+r_{j})-1\right)\ln\frac{\bar{F}(x_{ij})}{\bar{F}(\mu )}\right]{\text{I}}_{\left[x_{i1}>\mu \right]},$$ (6)

where $w_{i,\gamma }={\gamma (1\;-\;\gamma )}^{t-i}$ is the weight of log-likelihood function corresponding to *i*th batch sample and γ is a smoothing parameter. Similar to MLEs, the weighted maximum log-likelihood estimators (WMLE) can be obtained by maximizing $wl_{t}(\mu ,\lambda )$. It is clear that $wl_{t}(\mu ,\lambda )$ is an increasing function with respect to μ for $\mu \leq \min(x_{11},x_{21},\ldots ,x_{t1})$. Thus, the WMLE of μ, denoted by ${\tilde{\mu }}_{t}$, is obtained as ${\tilde{\mu }}_{t}=\min({\hat{\mu }}_{1},\ldots ,{\hat{\mu }}_{t})$, where ${\hat{\mu }}_{i}$ (1≤i≤t) is the MLE of μ based on the *i*th batch sample. Also, WMLE of λ, denoted by $\tilde{\lambda }$ is obtained by the equation


$$\frac{\partial wl_{t}({\tilde{\mu }}_{t},\lambda )}{\partial \lambda }=\sum_{i=1}^{t}w_{i,\gamma }\left[\frac{m}{\lambda }+\sum_{j=1}^{m}(1+r_{j})\ln\frac{\bar{F}(x_{ij})}{\bar{F}({\tilde{\mu }}_{t})}\right]=0.$$

Solving the above equation immediately implies that


$${\tilde{\lambda }}_{t}=m\sum_{i=1}^{t}w_{i,\gamma }{\left[-\sum_{i=1}^{t}w_{i,\gamma }\sum_{j=1}^{m}(1+r_{j})\ln\frac{\bar{F}(x_{ij})}{\bar{F}({\tilde{\mu }}_{t})}\right]}^{-1}.$$

Then, the WL charting statistic can be obtained as


$$WL_{t}=wl_{t}({\tilde{\mu }}_{t},{\tilde{\lambda }}_{t})-wl_{t}(\mu_{0},\lambda_{0})$$

$$=\left\{ \begin{array}{c} \infty ,\;\;\;\;{\tilde{\mu }}_{t}<\mu_{0},\\ \sum_{i=1}^{t}w_{i,\gamma }\left[m\left(\frac{\lambda 0}{{\tilde{\lambda }}_{t}}-\ln\frac{\lambda 0}{{\tilde{\lambda }}_{t}}-1\right)+n\lambda_{0}\ln\frac{\bar{F}(\mu_{0})}{\bar{F}({\tilde{\mu }}_{t})}\right],\;{\tilde{\mu }}_{t}>\mu_{0}. \end{array}\right.$$ (7)

The WL control chart generates signals when *WL*_*t*_>*h*, where the value of *h* is selected to attain a specified expected ${\text{ARL}}_{0}$.

By a simple algebraic calculation, ${\tilde{\lambda }}_{t}$ can be rewritten as


$${\tilde{\lambda }}_{t}=m\sum_{i=1}^{t}w_{i,\gamma }{\left[\sum_{i=1}^{t}w_{i,\gamma }\left(\frac{m}{{\hat{\lambda }}_{i}}-n\ln\frac{\bar{F}({\hat{\mu }}_{i})}{\bar{F}({\tilde{\mu }}_{t})}\right)\right]}^{-1},$$

where ${\hat{\mu }}_{i}$ and ${\hat{\lambda }}_{i}$ were defined in (2). Therefore, $\left({\tilde{\mu }}_{t},{\tilde{\lambda }}_{t}\right)$ is a function of $\left({\hat{\mu }}_{1},\ldots ,{\hat{\mu }}_{t},{\hat{\lambda }}_{1},\ldots ,{\hat{\lambda }}_{t}\right)$. On the other hand, based on the discussions in Subsect 1.1, the distribution of $\left({\hat{\mu }}_{1},\ldots ,{\hat{\mu }}_{t},{\hat{\lambda }}_{1},\ldots ,{\hat{\lambda }}_{t}\right)$ does not depend on r which implies that the distribution of $\left({\tilde{\mu }}_{,}{\tilde{\lambda }}_{t}\right)$ is free of the censoring scheme r. Hence, the WL control chart for the LTPHR model under progressive Type-II censoring is free of the censoring scheme and only depends on *m* and *n*.

## 2 Monte Carlo algorithm for implementation of the proposed control charts

As discussed in our proposed control charts, it is essential to determine the control limit *h* in order to achieve a desired expected value ${\text{ARL}}_{0}$. To facilitate this, we propose an algorithm specifically for designing the EWMA-LR control chart. Similar algorithms can be applied to other control charts as well. It is necessary to mention that, after obtaining the suitable control limit by Algorithm 2.1 and fixing *h* as a desirable value, we can compute the OOC ARL for the given shifted parameters $\mu_{1}$ and $\lambda_{1}$ using Steps (i)-(vii) of Algorithm 2.1. For this purpose, it is enough to replace $\mu_{0}$ and $\lambda_{0}$ by the respective values $\mu_{1}$ and $\lambda_{1}$ in Steps (ii) and (iii).

**Algorithm 2.1. Given the life test sample size *n*, the number of observations *m*, the baseline survival function $\bar{F}(x)$, the IC values parameters $\mu_{0}$ and $\lambda_{0}$, the smoothing parameter γ, the nominal ${\text{ARL}}_{0}$, the number of replications of the Monte Carlo simulations *M*, and a starting value *h*,**

(i) Set *i* = 1.
(ii) Randomly generate *B*_1_ and *B*_2_ such that $B_{1}\sim \text{B}(n\lambda_{0},1)$ and $B_{2}\sim \chi_{2m-2}^{2}$.
(iii) Compute $\bar{F}({\hat{\mu }}_{i})=\bar{F}(\mu_{0})B_{1}$ and ${\hat{\lambda }}_{i}=2m\lambda_{0}/B_{2}$.
(iv) Calculate *EL*_*i*_ by Eq (4).
(v) If *EL*_*i*_<*h*, set *i* = *i* + 1 and repeat Steps (ii)-(iv). And if *EL*_*i*_>*h*, the process is declared to be out of control (OOC) at the *i*th sample for the first time and set *RL* = *i*.
(vi) Repeat steps (i)-(v) for *M* times to obtain $RL_{1},\ldots ,RL_{M}$.
(vii) Compute $\text{ARL}=\sum_{j=1}^{M}RL_{j}/M$. Note that SDRL is also defined as $\text{SDRL}=\sum_{j=1}^{M}{\left(RL_{j}-ARL\right)}^{2}/(M-1)$.
(viii) Compare the obtained value of ARL in Step (vii) to the nominal ${\text{ARL}}_{0}$. If the difference between these two values is negligible, record the starting control limit *h*.
(ix) When the obtained ARL is greater (smaller) than ${\text{ARL}}_{0}$, decrease (increase) the control limit *h* to an appropriate value and repeat Step (i)-(viii) until ARL becomes fairly closer to the target nominal value of ${\text{ARL}}_{0}$. When we get the control limit under a set of parameters, it can be used to plot the control chart of the phase-II process.

## 3 Simulation study

In this section, we employ a Monte Carlo simulation to assess the performance of the proposed control charts presented in this paper. All computations are carried out using the R software.

We mainly evaluate the performance of the proposed control charts based on the ARL and SDRL. We first determined the control limits for the specified values: $\bar{F}(\mu_{0})=0.8$, $\lambda_{0}=1$, ${\text{ARL}}_{0}=370$, γ∈{0.05,0.1}, along with various values of *n* and *m*. The simulation results are summarized in Table 1. All results presented in this paper are derived from *M* = 20000 replications.

**Table 1:** Means and control limits of four proposed control charts for $\bar{F}(\mu_{0})=0.8$, $\lambda_{0}=1$, ${\text{ARL}}_{0}=370$ and different values of *n*, *m*, and γ.

n	m	EWMA-LR			EWMA-Max-MLE		EWMA-MD			WL	
		*EL*_0_	γ		γ		*EMD*_0_	γ		γ	
			0.05	0.1	0.05	0.1		0.05	0.1	0.05	0.1
5	3	2.0271	2.6606	3.1162	1.3444	1.4857	0.6992	0.8249	0.9085	0.1141	0.2417
	4	1.8541	2.4297	2.8434	1.3446	1.4856	0.6241	0.7433	0.8229	0.1129	0.2398
	5	1.7666	2.3145	2.7052	1.3442	1.4850	0.5746	0.6887	0.7652	0.1121	0.2386
10	3	2.0272	2.6621	3.1148	1.3445	1.4852	0.5992	0.7075	0.7776	0.1143	0.2420
	4	1.8549	2.4301	2.8421	1.3444	1.4852	0.5241	0.6249	0.6908	0.1127	0.2397
	6	1.7132	2.2448	2.6225	1.3444	1.4855	0.4394	0.5283	0.5868	0.1120	0.2379
	10	1.6193	2.1200	2.4731	1.3444	1.4852	0.3586	0.4336	0.4831	0.1112	0.2365
15	3	2.0275	2.6614	3.1179	1.3443	1.4852	0.5659	0.6707	0.7385	0.1142	0.2423
	4	1.8542	2.4291	2.8405	1.3443	1.4850	0.4906	0.5878	0.6515	0.1129	0.2397
	6	1.7137	2.2438	2.6221	1.3443	1.4852	0.4059	0.4909	0.5467	0.1118	0.2375
	10	1.6193	2.1195	2.4769	1.3445	1.4853	0.3253	0.3952	0.4412	0.1111	0.2367
	12	1.5969	2.0905	2.4431	1.3445	1.4853	0.3018	0.3669	0.4099	0.1112	0.2364

We use the zero-state ARL. The zero-state ARL refers to the expected number of samples collected before a control chart signals an OOC condition, assuming that any process shift occurs at the start of the monitoring period. At a specific time τ, let CED(τ) represent the expected number of samples until an OOC signal is triggered, given that no false alarms have occurred prior to this point. In this scenario, a process shift is assumed to happen at time τ. It is important to note that the zero-state ARL is equivalent to *CED*(1), while the steady-state ARL is defined as the limit of CED(τ) as τ tends to infinity, provided that such a limit exists. For more details, we refer to [30–32].

To evaluate the performance of the proposed control charts in terms of OOC ARL and SDRL, we must consider certain shifted parameters. Specifically, when the process goes OOC, the parameters shift from the IC parameters $\mu_{0}$ and $\lambda_{0}$ to the OOC parameters $\mu_{1}$ and $\lambda_{1}$. Therefore, in view of the proposed charting statistics in this paper, we consider shift sizes $\delta_{1}$ and $\delta_{2}$ as


$$\delta_{1}=\lambda_{0}\ln\frac{\bar{F}(\mu_{0})}{\bar{F}(\mu_{1})},\;\delta_{2}=\frac{\lambda_{0}}{\lambda_{1}}.$$

It is clear that $\left(\delta_{1},\delta_{2})=(0,1\right)$ indicates the IC process. We obtained the ARL and SDRL of the control charts for (n,m)∈{(5, 3),(5, 4),(10, 4),(10, 6),(15, 6),(15, 12)} and some values of $\delta_{1}$ and $\delta_{2}$. The results are reported in Tables 2, 3, 4, 5, 6 and 7.

**Table 2:** ARL and SDRL (in parentheses) values of the proposed control charts for *n* = 5, *m* = 3 and different values of γ, $\delta_{1}$ and $\delta_{2}$.

$\delta_{1}$	$\delta_{2}$	γ=0.05				γ=0.1			
		EWMA-LR	EWMA-Max-MLE	EWMA-MD	WL	EWMA-LR	EWMA-Max-MLE	EWMA-MD	WL
-0.2	0.8	1.40 (0.76)	1.40 (0.75)	9231.49 (9259.03)	1.24 (0.58)	1.41 (0.75)	1.40 (0.75)	9830.79 (9805.91)	1.25 (0.59)
-0.2	0.9	1.49 (0.86)	1.50 (0.87)	9160.82 (9117.10)	1.29 (0.65)	1.49 (0.86)	1.49 (0.85)	5294.39 (5290.88)	1.31 (0.69)
-0.2	1.0	1.58 (0.95)	1.57 (0.96)	4493.63 (4538.45)	1.36 (0.76)	1.58 (0.94)	1.59 (0.94)	2224.11 (2208.99)	1.37 (0.78)
-0.2	1.1	1.67 (1.06)	1.67 (1.06)	1654.50 (1666.61)	1.40 (0.82)	1.67 (1.05)	1.67 (1.08)	895.86 (902.53)	1.42 (0.84)
-0.2	1.2	1.76 (1.16)	1.76 (1.15)	602.52 (616.68)	1.45 (0.89)	1.76 (1.18)	1.77 (1.17)	377.92 (381.64)	1.47 (0.93)
-0.2	1.3	1.86 (1.26)	1.86 (1.26)	238.49 (240.27)	1.48 (0.94)	1.86 (1.26)	1.86 (1.26)	176.52 (177.06)	1.52 (1.00)
-0.1	0.8	2.15 (1.57)	2.16 (1.58)	18506.08 (18537.06)	1.64 (1.15)	2.13 (1.55)	2.13 (1.56)	11897.98 (11908.23)	1.68 (1.22)
-0.1	0.9	2.32 (1.75)	2.34 (1.78)	10111.72 (10186.17)	1.76 (1.34)	2.34 (1.78)	2.35 (1.78)	4870.46 (4884.46)	1.82 (1.42)
-0.1	1.0	2.56 (1.99)	2.55 (1.95)	3611.57 (3637.48)	1.86 (1.50)	2.55 (1.99)	2.56 (1.99)	1793.70 (1807.13)	1.91 (1.55)
-0.1	1.1	2.74 (2.19)	2.74 (2.16)	1158.22 (1169.79)	1.94 (1.63)	2.74 (2.21)	2.73 (2.18)	674.43 (678.57)	2.03 (1.73)
-0.1	1.2	2.91 (2.36)	2.90 (2.32)	406.12 (412.49)	1.99 (1.73)	2.90 (2.32)	2.95 (2.39)	281.55 (279.73)	2.09 (1.83)
-0.1	1.3	3.10 (2.56)	3.12 (2.56)	167.55 (167.64)	2.03 (1.75)	3.10 (2.55)	3.10 (2.55)	133.10 (131.67)	2.13 (1.88)
0.0	0.8	243.35 (238.96)	884.44 (888.69)	825.84 (820.05)	13.16 (25.13)	264.64 (254.74)	753.94 (750.84)	1206.01 (1192.27)	22.52 (43.96)
0.0	0.9	338.65 (333.63)	697.16 (695.13)	641.98 (634.05)	61.28 (151.19)	339.39 (334.31)	633.66 (632.62)	720.92 (709.91)	118.08 (245.29)
0.0	1.0	370.80 (365.85)	369.58 (363.44)	370.42 (364.34)	371.46 (896.86)	370.34 (365.89)	371.85 (364.12)	371.32 (371.10)	371.12 (742.75)
0.0	1.1	331.49 (324.60)	175.14 (166.70)	193.32 (187.03)	62.89 (145.28)	343.43 (343.05)	188.93 (183.78)	189.52 (184.15)	88.37 (176.19)
0.0	1.2	262.13 (256.16)	88.86 (79.94)	103.21 (96.45)	17.52 (34.71)	278.32 (276.58)	99.85 (92.60)	100.36 (96.86)	26.78 (50.58)
0.0	1.3	183.86 (176.98)	51.34 (42.16)	60.40 (54.26)	9.14 (15.31)	206.65 (200.97)	56.81 (50.50)	58.85 (55.16)	12.71 (21.53)
0.1	0.8	42.70 (31.42)	18815.98 (18565.06)	52.93 (38.18)	2.06 (1.25)	57.05 (49.93)	6051.78 (6081.21)	83.48 (72.74)	2.32 (1.52)
0.1	0.9	48.86 (37.01)	4856.59 (4854.10)	53.92 (40.76)	2.14 (1.30)	67.67 (59.50)	2454.23 (2476.98)	77.97 (68.36)	2.44 (1.60)
0.1	1.0	51.92 (40.10)	1024.53 (1023.37)	48.19 (36.72)	2.15 (1.31)	70.27 (62.21)	773.15 (770.51)	61.73 (53.84)	2.47 (1.63)
0.1	1.1	48.90 (37.91)	268.07 (263.56)	39.06 (29.37)	2.14 (1.30)	67.81 (60.12)	257.94 (252.39)	45.95 (39.19)	2.45 (1.61)
0.1	1.2	44.50 (33.02)	99.92 (91.98)	30.49 (22.19)	2.12 (1.27)	59.40 (51.51)	106.08 (101.61)	33.87 (28.20)	2.40 (1.58)
0.1	1.2	44.50 (33.02)	99.92 (91.98)	30.49 (22.19)	2.12 (1.27)	59.40 (51.51)	106.08 (101.61)	33.87 (28.20)	2.40 (1.58)
0.1	1.3	38.34 (27.42)	51.47 (43.25)	23.90 (16.88)	2.05 (1.24)	49.83 (41.74)	54.85 (49.51)	25.01 (20.13)	2.27 (1.49)
0.2	0.8	18.32 (9.54)	451.50 (442.16)	19.24 (9.41)	2.45 (1.61)	21.86 (14.80)	549.24 (543.47)	22.55 (14.22)	2.54 (1.68)
0.2	0.9	19.67 (10.57)	188.32 (176.47)	19.77 (10.35)	1.46 (1.61)	24.21 (16.68)	253.65 (243.50)	22.80 (15.05)	1.57 (0.70)
0.2	1.0	20.07 (10.87)	86.11 (73.41)	19.19 (10.46)	1.47 (0.62)	24.42 (16.79)	114.08 (105.86)	21.61 (14.57)	1.57 (0.70)
0.2	1.1	19.69 (10.55)	47.26 (35.88)	17.56 (9.70)	1.47 (0.62)	24.08 (16.67)	58.71 (50.57)	19.06 (12.94)	1.57 (0.69)
0.2	1.2	18.70 (9.97)	30.53 (20.73)	15.60 (8.72)	1.45 (0.61)	22.51 (19.19)	35.15 (27.89)	16.27 (10.80)	1.55 (0.68)
0.2	1.3	17.37 (8.97)	21.94 (13.52)	13.64 (7.59)	1.44 (0.60)	20.45 (13.40)	24.15 (17.89)	13.75 (9.11)	1.53 (0.68)
0.3	0.8	11.23 (4.51)	36.16 (21.69)	11.40 (4.37)	1.22 (0.42)	12.11 (6.13)	53.27 (42.65)	11.66 (5.50)	1.27 (0.44)
0.3	0.9	11.78 (4.82)	27.53 (15.14)	11.71 (4.73)	1.24 (0.42)	12.72 (6.61)	36.10 (26.20)	11.90 (5.96)	1.28 (0.45)
0.3	1.0	11.95 (4.94)	21.34 (10.70)	11.53 (4.94)	1.24 (0.42)	12.97 (6.73)	25.62 (17.07)	11.66 (6.03)	1.29 (0.45)
0.3	1.1	11.77 (4.80)	17.26 (8.26)	11.03 (4.86)	1.24 (0.42)	12.85 (6.68)	19.18 (11.82)	10.97 (5.82)	1.28 (0.45)
0.3	1.2	11.38 (4.64)	14.31 (6.51)	10.21 (4.60)	1.23 (0.42)	12.36 (6.41)	14.97 (8.61)	10.10 (5.46)	1.27 (0.45)
0.3	1.3	10.95 (4.46)	12.15 (5.32)	9.40 (4.35)	1.22 (0.42)	11.62 (5.91)	12.36 (6.75)	9.11 (4.94)	1.27 (0.44)
0.4	0.8	8.10 (2.71)	13.65 (4.41)	8.16 (2.59)	1.06 (0.24)	8.17 (3.36)	14.99 (6.76)	7.79 (2.98)	1.10 (0.30)
0.4	0.9	8.32 (2.77)	12.13 (3.88)	8.29 (2.84)	1.06 (0.25)	8.49 (3.49)	12.85 (5.60)	7.94 (3.23)	1.11 (0.31)
0.4	1.0	8.46 (2.85)	10.91 (3.50)	8.25 (2.93)	1.07 (0.25)	8.53 (3.48)	11.17 (4.69)	7.88 (3.34)	1.10 (0.31)
0.4	1.1	8.34 (2.81)	9.85 (3.14)	7.99 (2.97)	1.07 (0.25)	8.50 (3.48)	9.81 (3.99)	7.60 (3.31)	1.11 (0.31)
0.4	1.2	8.19 (2.74)	8.91 (2.87)	7.58 (2.92)	1.06 (0.24)	8.26 (3.40)	8.70 (3.46)	7.19 (3.25)	1.11 (0.31)
0.4	1.3	7.90 (2.67)	8.14 (2.64)	7.15 (2.85)	1.06 (0.23)	7.95 (3.26)	7.80 (3.07)	6.70 (3.11)	1.10 (0.31)

**Table 3:** ARL and SDRL (in parentheses) values of the proposed control charts for *n* = 5, *m* = 4 and different values of γ, $\delta_{1}$ and $\delta_{2}$.

$\delta_{1}$	$\delta_{2}$	γ=0.05				γ=0.1			
		EWMA-LR	EWMA-Max-MLE	EWMA-MD	WL	EWMA-LR	EWMA-Max-MLE	EWMA-MD	WL
-0.2	0.8	1.40 (0.75)	13.35 (4.36)	7096.67 (7088.63)	1.25 (0.58)	1.40 (0.75)	1.40 (0.74)	8998.42 (8961.01)	1.27 (0.61)
-0.2	0.9	1.50 (0.84)	1.49 (0.86)	11192.93 (11172.51)	1.32 (0.68)	1.49 (0.86)	1.48 (0.84)	6644.80 (6675.07)	1.33 (0.70)
-0.2	1.0	1.58 (0.96)	1.58 (0.96)	5912.06 (5966.94)	1.37 (0.77)	1.58 (0.96)	1.58 (0.95)	2837.38 (2842.12)	1.38 (0.78)
-0.2	1.1	1.68 (1.07)	1.69 (1.08)	1872.53 (1894.74)	1.43 (0.86)	1.68 (1.08)	1.66 (1.05)	990.63 (992.44)	1.44 (0.85)
-0.2	1.2	1.77 (1.16)	1.77 (1.17)	544.27 (550.58)	1.45 (0.89)	1.76 (1.46)	1.76 (1.16)	356.75 (358.11)	1.50 (0.93)
-0.2	1.3	1.88 (1.28)	1.87 (1.28)	188.29 (188.35)	1.51 (0.95)	1.86 (1.26)	1.87 (1.26)	147.40 (146.44)	1.52 (0.98)
-0.1	0.8	2.15 (1.58)	2.14 (1.56)	14461.13 (14496.56)	1.67 (1.18)	2.14 (1.57)	2.15 (1.57)	10313.22 (10304.99)	1.72 (1.24)
-0.1	0.9	2.37 (1.74)	2.36 (1.78)	11794.69 (11820.94)	1.80 (1.37)	2.34 (1.77)	2.36 (1.78)	5594.67 (5582.03)	1.86 (1.42)
-0.1	1.0	2.54 (2.00)	2.56 (2.01)	4412.62 (4428.41)	1.91 (1.53)	2.54 (1.95)	2.53 (1.96)	2092.16 (2093.71)	1.96 (1.59)
-0.1	1.1	2.71 (2.16)	2.76 (2.18)	1229.85 (1237.30)	2.00 (1.65)	2.73 (2.14)	2.72 (2.16)	706.85 (708.13)	2.07 (1.73)
-0.1	1.2	2.94 (2.40)	2.91 (2.36)	358.98 (363.89)	2.03 (1.69)	2.96 (2.39)	2.91 (2.36)	256.46 (257.45)	2.11 (1.79)
-0.1	1.3	3.12 (2.60)	3.13 (2.54)	130.22 (128.68)	2.05 (1.72)	3.10 (2.52)	3.13 (2.57)	109.21 (107.12)	2.14 (1.83)
0.0	0.8	201.78 (194.33)	649.69 (649.27)	622.10 (612.55)	10.39 (17.57)	222.50 (215.39)	581.46 (574.70)	914.07 (906.95)	16.55 (29.45)
0.0	0.9	315.47 (309.01)	650.28 (651.56)	610.06 (605.00)	47.98 (110.44)	328.47 (323.51)	589.84 (590.40)	685.04 (681.66)	87.69 (177.91)
0.0	1.0	370.12 (363.28)	371.37 (362.31)	369.67 (362.76)	369.41 (879.20)	370.66 (367.80)	370.22 (366.59)	369.61 (367.22)	371.11 (716.98)
0.0	1.1	320.95 (312.83)	167.21 (159.33)	181.72 (175.38)	49.86 (109.87)	332.86 (326.10)	186.11 (179.22)	178.74 (174.90)	75.29 (143.25)
0.0	1.2	223.75 (215.94)	80.06 (70.73)	89.12 (82.20)	14.17 (25.74)	242.16 (237.68)	89.06 (83.41)	88.49 (84.06)	20.86 (37.24)
0.0	1.3	137.53 (128.69)	44.34 (35.13)	49.17 (42.67)	7.50 (11.34)	242.16 (237.68)	48.77 (42.60)	48.62 (44.40)	10.07 (15.61)
0.1	0.8	34.90 (24.04)	10430.34 (10309.96)	45.13 (31.19)	2.10 (1.23)	46.96 (39.68)	3975.06 (3959.80)	68.02 (58.04)	2.36 (1.50)
0.1	0.9	42.85 (31.39)	4448.06 (4518.09)	49.74 (36.74)	2.20 (1.29)	57.05 (48.72)	2250.44 (2261.14)	70.10 (60.56)	2.49 (1.58)
0.1	1.0	44.84 (33.19)	1028.24 (1044.84)	45.34 (33.66)	2.23 (1.31)	62.16 (53.83)	759.90 (746.38)	57.38 (49.77)	2.56 (1.62)
0.1	1.1	42.99 (31.64)	257.00 (252.55)	35.91 (25.99)	2.21 (1.30)	49.12 (41.63)	249.78 (246.16)	42.29 (35.63)	2.52 (1.61)
0.1	1.2	37.31 (26.53)	91.22 (82.32)	27.43 (19.43)	2.14 (1.26)	49.38 (42.08)	96.87 (92.07)	29.70 (23.97)	2.43 (1.54)
0.1	1.3	30.97 (20.91)	44.58 (35.98)	20.60 (13.93)	2.07 (1.21)	39.18 (31.87)	46.78 (41.08)	21.25 (16.56)	2.32 (1.46)
0.2	0.8	15.70 (7.71)	342.34 (328.84)	17.37 (8.14)	1.47 (0.60)	18.00 (11.40)	428.08 (415.30)	19.71 (11.94)	1.56 (0.68)
0.2	0.9	17.24 (8.59)	181.19 (166.78)	18.42 (9.34)	1.49 (0.61)	20.20 (13.23)	239.83 (231.39)	20.98 (13.44)	1.60 (0.70)
0.2	1.0	17.60 (8.86)	85.44 (72.72)	17.92 (9.40)	1.51 (0.62)	21.08 (13.92)	112.35 (103.31)	19.96 (13.08)	1.60 (0.70)
0.2	1.1	17.21 (8.67)	46.22 (34.40)	16.38 (8.77)	1.50 (0.62)	20.45 (13.29)	57.05 (49.17)	17.41 (11.37)	1.59 (0.70)
0.2	1.2	16.10 (7.94)	29.04 (19.61)	14.30 (7.69)	1.48 (0.61)	18.72 (11.96)	21.91 (15.80)	14.58 (9.39)	1.58 (0.69)
0.2	1.3	14.79 (7.20)	20.52 (12.48)	12.18 (6.57)	1.46 (0.59)	16.61 (10.21)	33.43 (26.47)	12.16 (7.70)	1.56 (0.68)
0.3	0.8	9.85 (3.71)	34.24 (20.09)	10.55 (3.86)	1.23 (0.42)	10.28 (4.83)	426.58 (413.55)	10.59 (4.76)	1.28 (0.45)
0.3	0.9	10.46 (3.99)	27.31 (14.95)	10.97 (4.30)	1.25 (0.43)	10.98 (5.28)	36.03 (26.33)	11.06 (5.31)	1.30 (0.46)
0.3	1.0	10.61 (4.09)	21.27 (10.72)	10.86 (4.51)	1.25 (0.44)	11.30 (5.51)	25.30 (16.62)	10.88 (5.45)	1.30 (0.46)
0.3	1.1	10.44 (3.97)	17.12 (8.23)	10.28 (4.36)	1.25 (0.44)	11.10 (5.37)	18.86 (11.53)	10.21 (5.22)	1.31 (0.46)
0.3	1.2	10.02 (3.86)	14.09 (6.45)	9.45 (4.14)	1.24 (0.43)	11.53 (5.09)	11.88 (6.35)	9.23 (4.83)	1.29 (0.45)
0.3	1.3	9.43 (3.61)	11.84 (5.16)	8.56 (3.85)	1.23 (0.42)	9.81 (6.67)	11.89 (6.39)	8.24 (4.32)	1.28 (0.45)
0.4	0.8	7.16 (2.26)	13.46 (4.33)	7.58 (2.31)	1.06 (0.23)	7.08 (2.69)	14.66 (6.65)	7.18 (2.63)	1.10 (0.30)
0.4	0.9	7.46 (2.36)	12.13 (3.91)	7.80 (2.53)	1.06 (0.24)	7.39 (2.83)	12.84 (5.54)	7.44 (2.88)	1.11 (0.31)
0.4	1.0	7.51 (2.35)	10.91 (3.51)	7.80 (2.69)	1.06 (0.24)	7.52 (2.85)	11.13 (4.66)	7.43 (2.01)	1.12 (0.32)
0.4	1.1	7.44 (2.34)	9.80 (3.17)	7.51 (2.68)	1.06 (0.24)	7.43 (2.83)	9.73 (3.98)	7.09 (2.99)	1.11 (0.32)
0.4	1.2	7.25 (2.29)	8.87 (2.83)	7.09 (2.63)	1.06 (0.24)	7.17 (2.73)	8.60 (3.45)	6.65 (2.89)	1.11 (0.31)
0.4	1.3	6.99 (2.25)	8.06 (2.60)	6.59 (2.54)	1.05 (0.23)	6.88 (2.64)	7.68 (3.06)	6.15 (2.76)	1.10 (0.30)

**Table 4:** ARL and SDRL (in parentheses) values of the proposed control charts for *n* = 10, *m* = 4 and different values of γ, $\delta_{1}$ and $\delta_{2}$.

$\delta_{1}$	$\delta_{2}$	γ=0.05				γ=0.1			
		EWMA-LR	EWMA-Max-MLE	EWMA-MD	WL	EWMA-LR	EWMA-Max-MLE	EWMA-MD	WL
-0.2	0.8	1.09 (0.31)	1.09 (0.31)	53.27 (39.06)	1.06 (0.26)	1.09 (0.31)	1.09 (0.32)	82.28 (71.25)	1.06 (0.26)
-0.2	0.9	1.12 (0.37)	1.12 (0.37)	109.84 (95.63)	1.08 (0.30)	1.12 (0.37)	1.12 (0.37)	172.80 (163.23)	1.09 (0.31)
-0.2	1.0	1.15 (0.41)	1.15 (0.42)	169.38 (157.99)	1.10 (0.35)	1.15 (0.42)	1.16 (0.43)	213.56 (205.79)	1.11 (0.36)
-0.2	1.1	1.19 (0.47)	1.19 (0.48)	163.19 (164.66)	1.12 (0.39)	1.20 (0.50)	1.20 (0.48)	170.31 (165.66)	1.14 (0.40)
-0.2	1.2	1.23 (0.53)	1.22 (0.52)	115.35 (109.34)	1.16 (0.44)	1.22 (0.53)	1.27 (0.58)	109.00 (104.58)	1.16 (0.45)
-0.2	1.3	1.27 (0.58)	1.27 (0.58)	71.86 (66.65)	1.17 (0.46)	1.28 (0.60)	1.27 (0.59)	67.31 (64.53)	1.17 (0.46)
-0.1	0.8	1.39 (0.75)	1.40 (0.76)	471.72 (456.14)	1.24 (0.57)	1.40 (0.75)	1.40 (0.75)	877.10 (869.45)	1.27 (0.61)
-0.1	0.9	1.49 (0.85)	1.47 (0.86)	1292.16 (1291.53)	1.31 (0.69)	1.48 (0.84)	1.49 (0.86)	1362.37 (1367.49)	1.33 (0.71)
-0.1	1.0	1.57 (0.96)	1.59 (0.96)	1325.35 (1217.77)	1.37 (0.76)	1.58 (0.96)	1.58 (0.97)	933.74 (929.31)	1.40 (0.80)
-0.1	1.1	1.67 (1.06)	1.68 (1.06)	703.91 (710.72)	1.43 (0.86)	1.67 (1.06)	1.67 (1.06)	462.91 (459.46)	1.44 (0.86)
-0.1	1.2	1.75 (1.14)	1.77 (1.14)	296.74 (300.81)	1.47 (0.90)	1.77 (1.17)	1.77 (1.16)	212.25 (212.04)	1.50 (0.94)
-0.1	1.3	1.86 (1.27)	1.85 (1.27)	130.61 (129.83)	1.50 (0.95)	1.87 (1.28)	1.86 (1.28)	103.64 (102.19)	1.53 (0.98)
0.0	0.8	202.22 (196.85)	639.19 (632.82)	224.65 (208.01)	10.27 (17.26)	223.66 (217.17)	570.24 (567.00)	380.91 (371.87)	16.43 (28.77)
0.0	0.9	316.76 (310.87)	653.88 (642.68)	396.92 (388.35)	46.42 (107.63)	323.88 (318.69)	586.72 (586.33)	504.27 (495.96)	89.33 (182.57)
0.0	1.0	370.34 (371.91)	371.13 (365.76)	369.73 (362.53)	369.39 (368.19)	370.72 (370.34)	369.97 (364.47)	371.12 (368.90)	370.24 (707.68)
0.0	1.1	320.01 (313.69)	166.67 (156.19)	225.29 (220.46)	48.16 (106.63)	326.37 (320.60)	179.92 (173.24)	205.15 (203.55)	74.62 (140.38)
0.0	1.2	224.53 (218.83)	80.04 (70.43)	117.14 (113.45)	14.13 (25.80)	240.19 (232.78)	88.53 (82.08)	107.81 (105.98)	21.27 (37.39)
0.0	1.3	139.10 (130.66)	44.51 (35.55)	64.13 (59.60)	7.54 (11.58)	157.98 (153.53)	48.74 (42.36)	59.00 (56.31)	10.24 (15.82)
0.1	0.8	15.74 (7.64)	339.29 (323.96)	26.65 (15.73)	1.46 (0.74)	18.04 (11.22)	427.61 (418.40)	32.97 (23.99)	1.56 (0.68)
0.1	0.9	17.24 (8.60)	180.14 (166.23)	33.35 (21.88)	1.49 (0.61)	20.14 (12.90)	238.02 (233.08)	42.39 (33.36)	1.60 (0.70)
0.1	1.0	17.74 (9.03)	86.21 (72.52)	35.77 (24.83)	1.50 (0.62)	21.08 (13.72)	113.27 (104.54)	43.98 (36.13)	1.61 (0.71)
0.1	1.1	17.13 (8.68)	46.58 (35.03)	32.56 (22.82)	1.49 (0.61)	20.37 (13.33)	57.05 (49.86)	37.53 (30.73)	1.60 (0.70)
0.1	1.2	16.26 (8.07)	29.40 (19.71)	26.91 (18.91)	1.47 (0.60)	18.54 (11.82)	33.04 (26.40)	28.97 (23.30)	1.57 (0.69)
0.1	1.3	14.75 (7.17)	20.54 (12.63)	21.19 (14.63)	1.45 (0.59)	16.42 (10.10)	21.74 (15.84)	21.75 (17.07)	1.56 (0.68)
0.2	0.8	7.14 (2.26)	13.40 (4.33)	12.34 (5.13)	1.06 (0.23)	7.06 (2.67)	14.70 (6.57)	12.49 (6.37)	1.10 (0.30)
0.2	0.9	7.44 (2.34)	12.11 (3.92)	13.77 (6.18)	1.06 (0.24)	7.37 (2.81)	12.79 (5.51)	14.31 (7.94)	1.12 (0.32)
0.2	1.0	7.53 (2.36)	10.97 (3.55)	14.39 (6.93)	1.06 (0.25)	7.47 (2.83)	11.13 (4.65)	14.99 (8.85)	1.11 (0.32)
0.2	1.1	7.45 (2.34)	9.86 (3.20)	14.10 (7.03)	1.07 (0.25)	7.37 (2.78)	9.79 (4.04)	14.45 (8.77)	1.11 (0.32)
0.2	1.2	7.25 (2.28)	8.90 (2.90)	12.95 (6.62)	1.06 (0.24)	7.12 (2.70)	8.61 (3.45)	12.98 (7.92)	1.11 (0.31)
0.2	1.3	6.97 (2.24)	8.03 (2.58)	11.55 (6.09)	1.06 (0.23)	6.86 (2.62)	7.68 (3.06)	11.35 (7.01)	1.11 (0.31)
0.3	0.8	4.67 (1.14)	6.06 (1.01)	8.04 (2.64)	1.00 (0.00)	4.35 (1.19)	5.55 (1.09)	7.57 (2.93)	1.00 (0.00)
0.3	0.9	4.77 (1.16)	5.83 (1.04)	8.65 (3.07)	1.00 (0.00)	4.45 (1.22)	5.30 (1.10)	8.20 (3.46)	1.00 (0.00)
0.3	1.0	4.81 (1.17)	5.61 (1.03)	8.88 (3.31)	1.00 (0.00)	4.51 (1.23)	5.08 (1.10)	8.51 (3.82)	1.00 (0.00)
0.3	1.1	4.77 (1.57)	5.38 (1.05)	8.79 (3.42)	1.00 (0.00)	4.49 (1.23)	5.86 (1.10)	8.38 (3.90)	1.00 (0.00)
0.3	1.2	4.70 (1.15)	5.15 (1.05)	8.40 (3.44)	1.00 (0.00)	4.39 (1.22)	4.64 (1.22)	7.93 (3.81)	1.00 (0.00)
0.3	1.3	4.60 (1.15)	4.96 (1.04)	7.82 (3.33)	1.00 (0.00)	4.29 (1.21)	4.45 (1.07)	7.34 (3.62)	1.00 (0.00)
0.4	0.8	3.54 (0.74)	4.17 (0.55)	5.98 (1.68)	1.00 (0.00)	3.23 (0.75)	3.68 (0.52)	5.46 (1.80)	1.00 (0.00)
0.4	0.9	3.60 (0.75)	4.08 (0.60)	6.33 (1.88)	1.00 (0.00)	3.28 (0.75)	3.59 (0.55)	5.79 (2.03)	1.00 (0.00)
0.4	1.0	3.61 (0.75)	3.99 (0.57)	6.45 (2.01)	1.00 (0.00)	3.30 (0.74)	3.51 (0.57)	5.94 (2.15)	1.00 (0.00)
0.4	1.1	3.60 (0.75)	3.81 (0.60)	6.40 (2.08)	1.00 (0.00)	3.27 (0.75)	3.43 (0.59)	5.90 (2.26)	1.00 (0.00)
0.4	1.2	3.56 (0.75)	3.81 (0.60)	6.19 (2.14)	1.00 (0.00)	3.24 (0.75)	3.35 (0.60)	5.68 (2.27)	1.00 (0.00)
0.4	1.3	3.51 (0.75)	3.72 (0.61)	5.88 (2.13)	1.00 (0.00)	3.18 (0.75)	3.26 (0.61)	5.41 (2.24)	1.00 (0.00)

**Table 5:** ARL and SDRL (in parentheses) values of the proposed control charts for *n* = 10, *m* = 6 and different values of γ, $\delta_{1}$ and $\delta_{2}$.

$\delta_{1}$	$\delta_{2}$	γ=0.05				γ=0.1			
		EWMA-LR	EWMA-Max-MLE	EWMA-MD	WL	EWMA-LR	EWMA-Max-MLE	EWMA-MD	WL
-0.2	0.8	1.09 (0.31)	1.09 (0.32)	38.83 (26.01)	1.06 (0.26)	1.09 (0.32)	1.09 (0.32)	53.88 (43.87)	1.06 (0.26)
-0.2	0.9	1.12 (0.37)	1.12 (0.36)	90.31 (74.99)	1.09 (0.31)	1.12 (0.37)	1.12 (0.37)	139.49 (130.08)	1.09 (0.31)
-0.2	1.0	1.16 (0.42)	1.16 (0.43)	157.27 (143.56)	1.11 (0.36)	1.15 (0.42)	1.16 (0.43)	201.70 (195.91)	1.11 (0.36)
-0.2	1.1	1.20 (0.48)	1.20 (0.48)	138.81 (129.39)	1.13 (0.40)	1.19 (0.48)	1.19 (0.48)	151.06 (145.81)	1.14 (0.40)
-0.2	1.2	1.23 (0.55)	1.23 (0.53)	81.91 (74.10)	1.16 (0.44)	1.23 (0.54)	1.24 (0.54)	83.78 (78.85)	1.17 (0.46)
-0.2	1.3	1.28 (0.59)	1.27 (0.59)	45.65 (39.49)	1.17 (0.46)	1.28 (0.60)	1.27 (0.58)	44.71 (40.39)	1.19 (0.48)
-0.1	0.8	1.40 (0.75)	1.40 (0.75)	286.88 (270.50)	1.26 (0.59)	1.39 (0.74)	1.40 (0.76)	510.16 (499.11)	1.27 (0.62)
-0.1	0.9	1.50 (0.87)	1.49 (0.86)	1360.02 (1363.18)	1.33 (0.70)	1.48 (0.84)	1.49 (0.85)	1459.88 (1453.00)	1.34 (0.72)
-0.1	1.0	1.58 (0.96)	1.58 (0.96)	1718.92 (1734.42)	1.40 (0.78)	1.58 (0.96)	1.58 (0.95)	1165.59 (1180.33)	1.42 (0.83)
-0.1	1.1	1.66 (1.07)	1.66 (1.05)	723.58 (730.12)	1.44 (0.86)	1.67 (1.05)	1.68 (1.08)	480.70 (484.44)	1.47 (0.89)
-0.1	1.2	1.77 (1.16)	1.77 (1.16)	222.94 (223.33)	1.48 (0.90)	1.76 (1.16)	1.76 (1.16)	169.51 (169.21)	1.51 (0.94)
-0.1	1.3	1.86 (1.25)	1.86 (1.27)	79.83 (76.08)	1.50 (0.92)	1.87 (1.25)	1.85 (1.25)	68.88 (66.24)	1.52 (0.96)
0.0	0.8	145.26 (136.62)	354.62 (345.05)	141.54 (127.07)	7.60 (11.12)	165.51 (160.35)	357.62 (349.55)	224.90 (214.99)	10.68 (16.74)
0.0	0.9	288.86 (283.87)	561.99 (561.59)	345.50 (334.06)	31.41 (65.32)	303.00 (300.05)	533.48 (531.86)	441.73 (438.42)	56.94 (109.70)
0.0	1.0	369.97 (363.28)	371.01 (362.77)	369.56 (362.69)	369.76 (852.28)	370.30 (367.40)	371.20 (364.38)	369.78 (363.80)	369.76 (693.19)
0.0	1.1	297.08 (290.42)	153.68 (144.93)	194.96 (189.72)	34.85 (73.04)	304.67 (300.61)	169.87 (162.97)	181.09 (177.68)	54.30 (100.75)
0.0	1.2	169.63 (159.90)	65.55 (56.08)	83.97 (77.21)	10.17 (16.46)	187.14 (182.52)	73.45 (67.66)	80.84 (77.28)	14.77 (23.69)
0.0	1.3	87.44 (79.00)	34.57 (26.32)	41.42 (35.89)	5.69 (7.30)	100.74 (96.13)	37.03 (30.99)	39.62 (35.82)	7.18 (9.78)
0.1	0.8	13.50 (6.18)	204.15 (191.73)	20.99 (11.31)	1.48 (0.59)	14.77 (8.60)	263.51 (254.70)	23.95 (15.98)	1.57 (0.67)
0.1	0.9	15.38 (7.33)	164.76 (151.60)	27.90 (16.99)	1.51 (0.61)	17.28 (10.66)	219.45 (212.33)	33.45 (25.12)	1.63 (0.70)
0.1	1.0	15.95 (7.66)	85.90 (73.74)	30.25 (20.11)	1.53 (0.62)	18.41 (11.36)	113.72 (104.87)	36.19 (28.68)	1.65 (0.71)
0.1	1.1	15.26 (7.20)	45.09 (33.87)	27.00 (17.92)	1.52 (0.62)	17.47 (10.61)	55.06 (47.54)	30.21 (23.60)	1.63 (0.70)
0.1	1.2	14.07 (6.65)	26.80 (17.80)	20.76 (13.58)	1.49 (0.60)	15.53 (9.20)	30.12 (23.19)	22.11 (16.66)	1.60 (0.69)
0.1	1.3	12.31 (5.65)	18.21 (10.66)	15.75 (9.96)	1.45 (0.59)	13.17 (7.62)	19.22 (13.63)	15.74 (11.39)	1.55 (0.67)
0.2	0.8	6.38 (1.92)	12.95 (4.18)	10.26 (3.90)	1.05 (0.23)	6.19 (2.21)	13.94 (6.18)	10.10 (4.72)	1.10 (0.30)
0.2	0.9	6.76 (2.02)	12.05 (3.90)	11.80 (4.89)	1.06 (0.24)	6.58 (2.33)	12.73 (5.47)	11.91 (6.07)	1.12 (0.32)
0.2	1.0	6.89 (2.04)	10.89 (3.52)	12.42 (5.45)	1.07 (0.25)	6.73 (2.38)	11.17 (4.70)	12.54 (6.82)	1.12 (0.32)
0.2	1.1	6.78 (2.04)	9.79 (3.15)	12.03 (5.50)	1.06 (0.24)	6.61 (2.33)	11.69 (6.93)	11.92 (6.67)	1.11 (0.32)
0.2	1.2	6.50 (1.97)	8.73 (2.87)	10.68 (5.10)	1.06 (0.24)	6.30 (2.26)	8.41 (3.26)	10.44 (5.98)	1.11 (0.31)
0.2	1.3	6.17 (1.92)	7.82 (2.56)	9.16 (4.44)	1.05 (0.22)	5.93 (2.17)	7.42 (2.93)	8.79 (4.99)	1.10 (0.31)
0.3	0.8	4.26 (1.00)	6.04 (1.02)	6.85 (2.11)	1.00 (0.00)	3.92 (1.02)	5.52 (1.09)	6.36 (2.29)	1.00 (0.00)
0.3	0.9	4.40 (1.02)	5.83 (1.02)	7.48 (2.44)	1.00 (0.00)	4.05 (1.05)	4.31 (1.10)	7.04 (2.72)	1.00 (0.00)
0.3	1.0	4.45 (1.02)	5.61 (1.04)	7.74 (2.64)	1.00 (0.00)	4.09 (1.05)	5.08 (1.09)	7.31 (2.99)	1.00 (0.00)
0.3	1.1	4.39 (1.01)	5.35 (1.04)	7.52 (2.70)	1.00 (0.00)	4.05 (1.06)	4.84 (1.09)	7.12 (3.06)	1.00 (0.00)
0.3	1.2	4.31 (1.02)	5.13 (1.03)	7.06 (2.66)	1.00 (0.00)	4.98 (1.05)	4.61 (1.09)	6.59 (2.95)	1.00 (0.00)
0.3	1.3	4.15 (1.01)	4.91 (1.04)	6.45 (2.54)	1.00 (0.00)	4.82 (1.04)	4.39 (1.07)	5.95 (2.69)	1.00 (0.00)
0.4	0.8	3.25 (0.67)	4.17 (0.55)	5.17 (1.37)	1.00 (0.00)	2.94 (0.67)	3.68 (1.51)	4.66 (1.40)	1.00 (0.00)
0.4	0.9	3.33 (0.67)	4.09 (0.56)	5.51 (1.52)	1.00 (0.00)	3.00 (0.60)	3.60 (0.55)	5.01 (1.60)	1.00 (0.00)
0.4	1.0	3.34 (0.66)	3.99 (0.57)	5.64 (1.62)	1.00 (0.00)	3.02 (0.66)	3.52 (0.57)	5.15 (1.72)	1.00 (0.00)
0.4	1.1	3.34 (0.67)	3.89 (0.59)	5.57 (1.69)	1.00 (0.00)	3.02 (0.66)	3.42 (0.58)	5.06 (1.78)	1.00 (0.00)
0.4	1.2	3.28 (0.68)	3.80 (0.60)	5.32 (1.72)	1.00 (0.00)	2.96 (0.67)	3.35 (0.60)	4.82 (1.78)	1.00 (0.00)

**Table 6:** ARL and SDRL (in parentheses) values of the proposed control charts for *n* = 15, *m* = 6 and different values of γ, $\delta_{1}$ and $\delta_{2}$.

$\delta_{1}$	$\delta_{2}$	γ=0.05				γ=0.1			
		EWMA-LR	EWMA-Max-MLE	EWMA-MD	WL	EWMA-LR	EWMA-Max-MLE	EWMA-MD	WL
-0.2	0.8	1.02 (0.16)	1.02 (0.15)	19.13 (9.84)	1.02 (0.13)	1.02 (0.15)	1.02 (0.15)	21.36 (13.81)	1.02 (0.13)
-0.2	0.9	1.04 (0.19)	1.04 (0.19)	30.66 (19.61)	1.02 (0.16)	1.04 (0.19)	1.04 (0.20)	38.05 (29.28)	1.03 (0.18)
-0.2	1.0	1.05 (0.24)	1.05 (0.23)	43.70 (32.12)	1.04 (0.20)	1.05 (0.23)	1.05 (0.24)	55.09 (46.89)	1.04 (0.20)
-0.2	1.1	1.07 (0.28)	1.07 (0.28)	46.14 (35.66)	1.05 (0.24)	1.07 (0.28)	1.07 (0.28)	53.74 (47.32)	1.05 (0.24)
-0.2	1.2	1.09 (0.31)	1.09 (0.31)	37.44 (29.01)	1.06 (0.25)	1.09 (0.31)	1.09 (0.31)	39.30 (34.30)	1.06 (0.25)
-0.2	1.3	1.11 (0.36)	1.11 (0.36)	26.91 (20.62)	1.08 (0.29)	1.11 (0.34)	1.11 (0.34)	26.99 (22.40)	1.08 (0.29)
-0.1	0.8	1.18 (0.46)	1.18 (0.47)	80.42 (66.29)	1.12 (0.38)	1.18 (0.47)	1.19 (0.47)	125.42 (115.47)	1.13 (0.38)
-0.1	0.9	1.22 (0.52)	1.23 (0.54)	277.11 (263.29)	1.17 (0.45)	1.24 (0.54)	1.23 (0.53)	388.89 (382.57)	1.17 (0.45)
-0.1	1.0	1.29 (0.61)	1.29 (0.62)	487.48 (484.90)	1.20 (0.50)	1.28 (0.60)	1.28 (0.60)	472.03 (471.34)	1.21 (0.52)
-0.1	1.1	1.34 (0.68)	1.34 (0.67)	320.07 (318.27)	1.23 (0.55)	1.34 (0.68)	1.34 (0.68)	267.76 (261.87)	1.24 (0.57)
-0.1	1.2	1.39 (0.74)	1.40 (0.75)	142.15 (139.15)	1.27 (0.62)	1.40 (0.75)	1.40 (0.75)	120.47 (118.24)	1.28 (0.64)
-0.1	1.3	1.46 (0.82)	1.46 (0.82)	63.00 (58.14)	1.28 (0.62)	1.46 (0.82)	1.46 (0.82)	56.89 (54.28)	1.30 (0.65)
0.0	0.8	143.44 (134.05)	360.60 (357.46)	104.32 (90.26)	7.53 (10.96)	166.25 (161.82)	363.71 (357.82)	163.06 (153.47)	10.72 (16.40)
0.0	0.9	288.43 (281.80)	556.44 (556.36)	287.83 (274.87)	31.02 (65.39)	299.95 (291.41)	520.99 (516.40)	386.95 (377.38)	57.77 (110.57)
0.0	1.0	371.33 (366.87)	371.28 (364.61)	369.01 (360.81)	369.85 (853.64)	370.63 (369.49)	370.66 (366.02)	370.24 (366.19)	370.27 (695.68)
0.0	1.1	294.84 (290.59)	152.32 (143.57)	215.48 (212.60)	34.34 (71.81)	304.13 (303.03)	168.96 (163.10)	196.38 (192.42)	56.30 (103.36)
0.0	1.2	168.39 (163.06)	65.94 (55.73)	95.12 (90.41)	10.38 (16.83)	184.35 (178.29)	73.66 (68.71)	87.89 (84.20)	14.61 (23.53)
0.0	1.3	88.44 (79.09)	34.48 (25.89)	45.72 (40.42)	5.68 (7.45)	100.98 (95.09)	36.56 (84.84)	42.80 (38.90)	7.20 (9.73)
0.1	0.8	8.64 (3.07)	30.39 (17.26)	18.42 (9.41)	1.24 (0.43)	8.76 (3.89)	41.47 (31.37)	20.36 (12.89)	1.30 (0.46)
0.1	0.9	9.34 (3.38)	26.65 (14.31)	24.70 (14.59)	1.26 (0.44)	9.64 (4.31)	34.42 (25.27)	29.19 (21.06)	1.32 (0.46)
0.1	1.0	9.59 (3.47)	21.27 (10.67)	28.15 (17.87)	1.27 (0.44)	9.95 (4.50)	25.60 (17.21)	33.74 (26.08)	1.32 (0.47)
0.1	1.1	9.42 (3.41)	16.94 (7.99)	26.64 (17.54)	1.26 (0.44)	9.65 (4.33)	18.63 (11.32)	29.73 (23.15)	1.32 (0.47)
0.1	1.2	8.89 (3.24)	13.57 (6.11)	21.19 (14.05)	1.25 (0.43)	9.03 (4.08)	14.03 (7.89)	22.40 (17.04)	1.30 (0.46)
0.1	1.3	8.20 (3.01)	11.14 (4.81)	16.02 (10.15)	1.23 (0.42)	8.18 (4.66)	11.00 (5.80)	16.09 (11.66)	1.28 (0.45)
0.2	0.8	4.25 (1.00)	6.03 (1.02)	9.43 (3.45)	1.00 (0.00)	3.90 (1.03)	5.50 (1.10)	9.11 (4.02)	1.00 (0.00)
0.2	0.9	4.39 (1.01)	5.83 (1.02)	10.96 (4.41)	1.00 (0.00)	4.07 (1.06)	5.30 (1.09)	10.87 (5.36)	1.00 (0.00)
0.2	1.0	4.43 (1.01)	5.60 (1.04)	11.80 (5.02)	1.00 (0.00)	4.10 (1.06)	5.08 (1.09)	11.83 (6.25)	1.00 (0.00)
0.2	1.1	4.39 (1.02)	5.37 (1.04)	11.50 (5.14)	1.00 (0.00)	4.06 (1.05)	4.85 (1.10)	11.55 (6.25)	1.00 (0.00)
0.2	1.2	4.29 (1.01)	5.14 (1.04)	10.47 (4.93)	1.00 (0.00)	3.96 (1.04)	4.62 (1.08)	10.29 (5.74)	1.00 (0.00)
0.2	1.3	4.15 (1.00)	4.90 (1.03)	9.10 (4.36)	1.00 (0.00)	3.82 (1.04)	4.38 (1.07)	8.76 (4.95)	1.00 (0.00)
0.3	0.8	2.92 (0.57)	3.72 (0.45)	6.36 (1.89)	1.00 (0.00)	2.63 (0.56)	3.17 (0.46)	5.86 (2.04)	1.00 (0.00)
0.3	0.9	2.98 (0.58)	3.65 (0.49)	7.02 (2.23)	1.00 (0.00)	2.68 (0.56)	3.12 (0.44)	6.55 (2.46)	1.00 (0.00)
0.3	1.0	2.99 (0.57)	3.57 (0.51)	7.34 (2.43)	1.00 (0.00)	2.68 (0.55)	3.07 (0.46)	6.91 (2.73)	1.00 (0.00)
0.3	1.1	2.98 (0.57)	3.50 (0.53)	7.26 (2.58)	1.00 (0.00)	2.68 (0.55)	3.01 (0.45)	6.80 (2.78)	1.00 (0.00)
0.3	1.2	2.94 (0.58)	3.43 (0.54)	6.87 (2.56)	1.00 (0.00)	2.65 (0.56)	2.96 (0.46)	6.44 (2.80)	1.00 (0.00)
0.3	1.3	2.88 (0.58)	3.35 (0.55)	6.33 (2.48)	1.00 (0.00)	2.60 (0.57)	2.90 (0.48)	5.83 (2.60)	1.00 (0.00)
0.4	0.8	2.26 (0.47)	2.94 (0.23)	4.85 (2.24)	1.00 (0.00)	2.00 (0.31)	2.47 (0.50)	4.36 (1.29)	1.00 (0.00)
0.4	0.9	2.30 (0.49)	2.91 (0.28)	5.21 (2.41)	1.00 (0.00)	2.01 (0.31)	2.41 (0.49)	4.70 (1.45)	1.00 (0.00)
0.4	1.0	2.31 (0.48)	2.88 (0.32)	5.38 (2.52)	1.00 (0.00)	2.02 (0.31)	2.36 (0.48)	4.88 (1.59)	1.00 (0.00)
0.4	1.1	2.31 (0.49)	2.84 (0.36)	5.34 (2.58)	1.00 (0.00)	2.01 (0.30)	2.31 (0.47)	4.84 (1.63)	1.00 (0.00)
0.4	1.2	2.28 (0.48)	2.80 (0.40)	5.11 (1.61)	1.00 (0.00)	2.00 (0.31)	2.28 (0.45)	4.65 (1.67)	1.00 (0.00)
0.4	1.3	2.24 (0.48)	2.76 (0.42)	4.84 (1.61)	1.00 (0.00)	1.98 (0.32)	2.25 (0.44)	4.37 (1.64)	1.00 (0.00)

**Table 7:** ARL and SDRL (in parentheses) values of the proposed control charts for *n* = 15, *m* = 12 and different values of γ, $\delta_{1}$ and $\delta_{2}$.

$\delta_{1}$	$\delta_{2}$	γ=0.05				γ=0.1			
		EWMA-LR	EWMA-Max-MLE	EWMA-MD	WL	EWMA-LR	EWMA-Max-MLE	EWMA-MD	WL
-0.2	0.8	1.02 (0.15)	1.02 (0.16)	12.08 (5.25)	1.02 (0.13)	1.02 (0.16)	1.02 (0.16)	12.27 (6.50)	1.02 (0.14)
-0.2	0.9	1.04 (0.19)	1.04 (0.20)	20.47 (11.34)	1.03 (0.17)	1.04 (0.20)	1.04 (0.20)	23.32 (15.86)	1.03 (0.18)
-0.2	1.0	1.05 (0.24)	1.05 (0.23)	30.45 (19.90)	1.04 (0.21)	1.06 (0.24)	1.05 (0.23)	37.97 (29.82)	1.04 (0.20)
-0.2	1.1	1.07 (0.28)	1.07 (0.28)	29.26 (19.20)	1.05 (0.23)	1.07 (0.28)	1.07 (0.27)	33.35 (26.51)	1.06 (0.25)
-0.2	1.2	1.09 (0.32)	1.09 (0.31)	20.36 (13.34)	1.06 (0.26)	1.09 (0.31)	1.09 (0.31)	20.97 (15.94)	1.06 (0.26)
-0.2	1.3	1.11 (0.35)	1.11 (0.35)	13.42 (8.26)	1.07 (0.27)	1.11 (0.35)	1.11 (0.35)	13.03 (9.14)	1.07 (0.29)
-0.1	0.8	1.18 (0.46)	1.18 (0.46)	37.91 (26.31)	1.12 (0.37)	1.18 (0.46)	1.18 (0.46)	49.35 (40.01)	1.13 (0.38)
-0.1	0.9	1.23 (0.53)	1.23 (0.53)	199.03 (185.85)	1.17 (0.45)	1.24 (0.54)	1.23 (0.53)	278.03 (267.90)	1.17 (0.46)
-0.1	1.0	1.29 (0.61)	1.29 (0.61)	558.75 (549.03)	1.21 (0.52)	1.29 (0.60)	1.29 (0.61)	549.03 (539.01)	1.22 (0.54)
-0.1	1.1	1.35 (0.69)	1.34 (0.67)	241.64 (238.66)	1.24 (0.57)	1.35 (0.68)	1.35 (0.68)	220.93 (217.67)	1.25 (0.58)
-0.1	1.2	1.40 (0.75)	1.40 (0.75)	66.00 (59.16)	1.27 (0.61)	1.40 (0.75)	1.40 (0.75)	63.37 (59.62)	1.28 (0.61)
-0.1	1.3	1.46 (0.82)	1.46 (0.82)	24.93 (19.57)	1.27 (0.60)	1.46 (0.81)	1.46 (0.82)	24.45 (20.69)	1.28 (0.62)
0.0	0.8	67.46 (57.92)	100.84 (90.36)	46.56 (34.68)	4.63 (5.18)	80.71 (74.86)	117.19 (111.48)	61.18 (52.38)	5.62 (6.75)
0.0	0.9	219.47 (211.28)	378.48 (374.32)	204.81 (191.30)	16.25 (28.98)	238.97 (235.32)	378.58 (372.96)	256.41 (257.61)	26.54 (46.77)
0.0	1.0	371.00 (367.44)	370.21 (366.81)	370.81 (364.40)	370.42 (833.40)	370.40 (364.42)	370.34 (367.93)	370.47 (367.33)	371.97 (683.12)
0.0	1.1	226.75 (220.88)	124.67 (114.56)	148.12 (141.37)	18.40 (33.57)	243.32 (239.00)	137.10 (130.82)	142.28 (137.86)	28.68 (49.72)
0.0	1.2	85.69 (77.17)	42.14 (33.30)	46.54 (39.95)	6.10 (7.90)	100.96 (95.61)	46.00 (39.97)	45.69 (41.61)	7.66 (10.16)
0.0	1.3	36.37 (28.65)	20.51 (13.48)	20.11 (15.04)	3.60 (3.73)	40.99 (35.28)	20.68 (15.57)	19.28 (15.88)	4.16 (4.48)
0.1	0.8	7.30 (2.53)	22.76 (11.89)	11.71 (5.03)	1.23 (0.42)	7.20 (2.96)	27.48 (18.49)	11.71 (6.07)	1.29 (0.45)
0.1	0.9	8.37 (2.89)	25.13 (13.51)	17.00 (8.67)	1.27 (0.44)	8.49 (3.58)	31.43 (22.43)	18.52 (11.72)	1.33 (0.47)
0.1	1.0	8.85 (3.06)	21.47 (10.78)	20.15 (11.28)	1.28 (0.45)	8.96 (3.85)	25.48 (17.00)	22.56 (15.42)	1.35 (0.48)
0.1	1.1	8.47 (2.89)	16.26 (7.60)	17.55 (9.98)	1.27 (0.45)	8.56 (3.64)	17.90 (10.80)	18.63 (12.42)	1.34 (0.47)
0.1	1.2	7.62 (2.69)	12.25 (5.44)	12.82 (7.00)	1.24 (0.43)	7.52 (3.22)	12.48 (6.81)	12.64 (8.16)	1.30 (0.46)
0.1	1.3	6.63 (2.41)	9.54 (3.98)	9.13 (4.80)	1.21 (0.41)	6.46 (2.73)	9.19 (4.62)	8.65 (5.28)	1.26 (1.44)
0.2	0.8	3.80 (0.90)	5.92 (1.01)	6.59 (2.08)	1.00 (0.00)	3.46 (0.90)	5.38 (1.11)	6.11 (2.25)	1.00 (0.00)
0.2	0.9	4.04 (0.91)	5.82 (1.03)	7.94 (2.72)	1.00 (0.00)	3.70 (0.94)	5.28 (1.10)	7.54 (3.08)	1.00 (0.00)
0.2	1.0	4.12 (0.90)	5.61 (1.03)	6.62 (3.15)	1.00 (0.00)	3.79 (0.93)	5.07 (1.10)	8.30 (3.64)	1.00 (0.00)
0.2	1.1	4.05 (0.91)	5.36 (1.04)	8.15 (3.11)	1.00 (0.00)	3.72 (0.93)	4.83 (1.10)	7.81 (3.55)	1.00 (0.00)
0.2	1.2	3.87 (0.93)	5.08 (1.04)	7.04 (3.84)	1.00 (0.00)	3.56 (0.94)	4.55 (1.08)	6.58 (3.10)	1.00 (0.00)
0.2	1.3	3.64 (0.93)	4.76 (1.02)	5.85 (2.41)	1.00 (0.00)	3.33 (0.93)	4.26 (1.06)	5.39 (2.57)	1.00 (0.00)
0.3	0.8	2.68 (0.52)	3.71 (0.46)	4.63 (1.22)	1.00 (0.00)	2.42 (0.55)	3.17 (0.46)	4.17 (1.24)	1.00 (0.00)
0.3	0.9	2.78 (0.51)	3.65 (0.49)	5.24 (1.41)	1.00 (0.00)	2.52 (0.53)	3.13 (0.45)	4.75 (1.50)	1.00 (0.00)
0.3	1.0	2.80 (0.50)	3.58 (0.51)	5.49 (1.55)	1.00 (0.00)	2.55 (0.53)	3.07 (0.45)	5.01 (1.64)	1.00 (0.00)
0.3	1.1	2.79 (0.51)	3.50 (0.53)	5.33 (1.59)	1.00 (0.00)	2.52 (0.54)	3.02 (0.46)	4.84 (1.67)	1.00 (0.00)
0.3	1.2	2.71 (0.53)	3.42 (0.54)	4.85 (1.55)	1.00 (0.00)	2.46 (0.55)	3.95 (0.47)	4.38 (1.61)	1.00 (0.00)
0.3	1.3	2.62 (0.55)	3.33 (0.54)	4.30 (1.48)	1.00 (0.00)	2.35 (0.56)	3.89 (0.48)	3.86 (1.50)	1.00 (0.00)
0.4	0.8	2.06 (0.33)	3.94 (0.23)	3.63 (0.82)	1.00 (0.00)	1.91 (0.28)	2.47 (0.50)	3.20 (0.83)	1.00 (0.00)
0.4	0.9	2.10 (0.36)	2.91 (0.28)	3.95 (0.92)	1.00 (0.00)	1.94 (0.24)	2.41 (0.49)	3.50 (0.93)	1.00 (0.00)
0.4	1.0	2.11 (0.36)	2.88 (0.32)	4.10 (0.97)	1.00 (0.00)	1.94 (0.23)	2.36 (0.48)	3.64 (0.98)	1.00 (0.00)
0.4	1.1	2.10 (0.36)	2.84 (0.37)	4.00 (1.01)	1.00 (0.00)	1.94 (0.24)	2.31 (0.47)	3.56 (1.01)	1.00 (0.00)
0.4	1.2	2.07 (0.35)	2.81 (0.40)	3.77 (1.03)	1.00 (0.00)	1.92 (0.28)	2.28 (0.46)	3.33 (1.02)	1.00 (0.00)
0.4	1.3	2.02 (0.35)	2.76 (0.43)	3.44 (1.03)	1.00 (0.00)	1.89 (0.32)	2.24 (0.44)	3.05 (1.00)	1.00 (0.00)

The summary of results derived from Tables 2, 3, 4, 5, 6 and 7 is listed below:

(i) When there is no shift in $\left(\mu_{0},\lambda_{0}\right)$, the simulated ${\text{ARL}}_{0}$ for all control charts is approximately equal to the nominal value 370. This indicates the stability of all the control charts proposed in this paper. Additionally, the SDRL for the EWMA-LR, EWMA-Max-MLE, and EWMA-MD control charts is close to${\text{ARL}}_{0}$, suggesting that the geometric distribution effectively approximates the IC run length distribution of these charts.

(ii) In certain scenarios, the EWMA-Max-MLE and EWMA-MD control charts exhibit ARL bias; specifically, the OOC ARL can exceed the nominal ${\text{ARL}}_{0}=370$. This outcome is less than ideal for practical applications.

(iii) In most instances, the EWMA-LR control chart outperforms both the EWMA-Max-MLE and EWMA-MD control charts with respect to the ARL and SDRL criteria.

(iv) The WL control chart provides the smallest OOC ARL and SDRL across all cases.

Overall, we recommend using the WL control chart for monitoring the LTPHR process, regardless of $\bar{F}(x)$, *n* and *m*.

## 4 Data analysis

We utilize the data sets presented in [33], which were gathered from a laboratory experiment involving two groups of male RFM strain mice. These mice were exposed to a radiation dose of 300 roentgens when they were 5 to 6 weeks old. The first group was housed in a conventional laboratory environment, while the second group was maintained in a germ-free environment. In his study, [33] identified two primary causes of death: thymic lymphoma and reticulum cell sarcoma, while categorizing all other causes into a single group. For our analysis, we focus on these two main groups: one in a conventional laboratory setting and the other in a germ-free environment.

[25] used these lifetime data by dividing by 1100 and got the data represented in Tables 8 and 9. They showed that the LTPHR models with the baseline survival function

**Table 8:** Lifetimes of mice lived in a conventional laboratory environment after receiving a radiation dose of 300 roentgens at an age of 5-6 weeks (divided by 1100).

0.0364	0.0381	0.0464	0.0564	0.1445	0.1482	0.1627	0.1718	0.1736	0.1800
0.1818	0.1873	0.1882	0.2000	0.2018	0.2073	0.2136	0.2227	0.2264	0.2273
0.2291	0.2327	0.2373	0.2409	0.2418	0.2545	0.2564	0.2882	0.2891	0.2945
0.3027	0.03100	0.3118	0.3236	0.3327	0.3482	0.3500	0.3627	0.3364	0.3700
0.3764	0.3818	0.3891	0.3918	0.3927	0.4009	0.4191	0.4200	0.4382	0.4500
0.4700	0.4700	0.4764	0.4773	0.4873	0.4991	0.5018	0.5036	0.5064	0.5073
0.5127	0.5155	0.5191	0.5327	0.5327	0.5400	0.5418	0.5500	0.5564	0.5627
0.5636	0.5645	0.5645	0.5655	0.5709	0.5736	0.5782	0.5845	0.5882	0.5882
0.5891	0.5900	0.5918	0.6009	0.6027	0.6055	0.6091	0.6236	0.6318	0.6336
0.6364	0.6409	0.6473	0.6482	0.6709	0.6800	0.6845	0.6918	0.6936	

**Table 9:** Lifetimes of mice lived in a germ-free environment after receiving a radiation dose of 300 roentgens at an age of 5-6 weeks (divided by 1100).

0.1236	0.1436	0.1745	0.1754	0.1764	0.1773	0.1836	0.1927	0.1955	0.2082
0.2091	0.2155	0.2182	0.2218	0.2236	0.2245	0.2318	0.2355	0.2727	0.2736
0.2918	0.3064	0.3418	0.3773	0.3827	0.3909	0.3945	0.4036	0.4409	0.4509
0.4809	0.4882	0.5136	0.5364	0.5509	0.5600	0.5609	0.5672	0.5800	0.5927
0.5955	0.5955	0.5981	0.6000	0.6018	0.6136	0.6173	0.6191	0.6282	0.6300
0.6327	0.6427	0.6673	0.6691	0.6700	0.6791	0.6836	0.6881	0.6909	0.6991
0.7064	0.7003	0.7273	0.7273	0.7336	0.7464	0.7500	0.7773	0.7791	0.7855
0.7891	0.7909	0.7909	0.7936	0.8018	0.8136	0.8273	0.8491	0.8564	0.8964
0.9227	0.9264								


$$\bar{F}(x)=\frac{\Gamma (1.2809,\frac{x}{0.3902(1-x)})}{\Gamma (1.2809,0)},\;0<x<1,$$

fit well to the data, such that


$$\Gamma (a,z)=\int_{z}^{\infty }t^{a-1}e^{-t}\text{d}t,\;z\geq 0,$$

is called an incomplete gamma function. The baseline survival function is called a gamma-uniform distribution introduced by [34]. For the data set in Table 8, the lifetime of mice lived in a conventional laboratory environment divided by 1100, they estimated $\hat{\mu }=0.0364$ ($\bar{F}(\hat{\mu })=0.9587$) and $\hat{\lambda }=0.5444$, and for the data set in Table 9, the lifetime of mice lived in a germ-free environment divided by 1100, they estimated $\hat{\mu }=0.1236$ ($\bar{F}(\hat{\mu })=0.8069$) and $\hat{\lambda }=0.2302$. Fig 1 presents the probability density function (PDF) and the cumulative distribution function (CDF) of two populations based on the estimated parameters.

**Fig 1:**
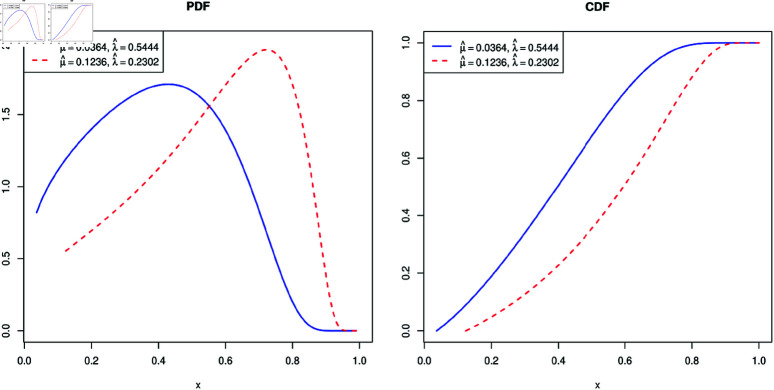
PDF and CDF of two populations based on the estimated parameters.

**Fig 2:**
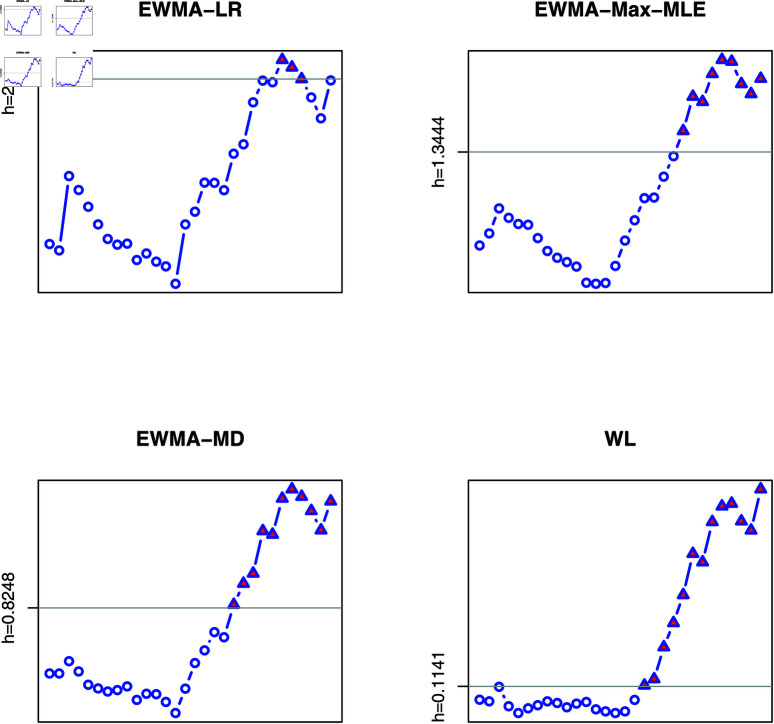
Monitoring of the lifetimes of mice after receiving a radiation dose of 300 roentgens at an age of 5-6 weeks.

Using such information, we reconstruct realistic data sets adopting a Monte Carlo simulation to illustrate how to use the proposed four control charts. We observe 15 values for $\left(\bar{F}({\hat{\mu }}_{0}),{\hat{\lambda }}_{0}\right)$ as the estimated parameters for a set of 15 samples with *n* = 5 and *m* = 3 (regardless of the censoring scheme r) with $\left(\bar{F}(\mu_{0}),\lambda_{0})=(0.9587,0.5444\right)$ as data from an apparently IC process, and another 15 observations for $\left(\bar{F}({\hat{\mu }}_{1}),{\hat{\lambda }}_{1}\right)$ as the estimated parameters for a set of 15 samples with *n* = 5 and *m* = 3 with $\left(\bar{F}(\mu_{1}),\lambda_{1})=(0.8069,0.2302\right)$ ($\delta_{1}=0.0939,\;\delta_{2}=2.3649$) as data from an apparently OOC process. The observed values are reported in Table 10. Based on these values and for γ=0.05, the control limits for the EWMA-LR, EWMA-Max-MLE, EWMA-MD, and WL control charts were obtained as 2.6606, 1.3444, 0.8248, and 0.1141, respectively. We display the corresponding control charts in Fig 2. We observe that the OOC signals for the EWMA-Max-MLE monitoring approach begin from the 22nd sample, while the EWMA-MD approach shows OOC signals starting from the 20th sample. In contrast, the WL monitoring method indicates OOC signals from the 18th sample onwards. The EWMA-LR monitoring approach, however, suggests that the process remains IC before the 25th sample, generating OOC signals only between the 25th and 27th observations. Notably, since the actual process shift begins from the 11th subgroup sample, this indicates that the WL control chart is capable of detecting shifts more effectively and earlier than the other monitoring charts.

**Table 10:** Estimated parameters for lifetimes of mice after receiving a radiation dose of 300 roentgens at an age of 5-6 weeks for *n* = 5, *m* = 3.

$\left(\bar{F}(\mu_{0}),\lambda_{0})=(0.9587,0.5444\right)$					
1	(0.7456,1.1896)	6	(0.9092,1.0856)	11	(0.6983,1.8897)
2	(0.9445,1.7504)	7	(0.8205,1.0194)	12	(0.7331,1.0680)
3	(0.6497,14.2823)	8	(0.7580,1.3304)	13	(0.5441,0.5153)
4	(0.6363,0.6088)	9	(0.5711,1.1599)	14	(0.7979,0.4903)
5	(0.6962,0.5533)	10	(0.7477,0.5552)	15	(0.2548,3.4421)
$\left(\bar{F}(\mu_{1}),\lambda_{1})=(0.8069,0.2302\right)$					
16	(0.5247,0.2333)	21	(0.5103,0.2364)	26	(0.7008,0.2447)
17	(0.1958,0.4072)	22	(0.1149,0.4908)	27	(0.5484,0.3720)
18	(0.6969,0.2390)	23	(0.6930,0.1623)	28	(0.6588,0.7616)
19	(0.5410,0.4283)	24	(0.4301,0.9075)	29	(0.6876,0.5021)
20	(0.2363,0.2409)	25	(0.5074,0.1809)	30	(0.2011,0.2430)

## 5 Conclusion

In this paper, we introduced four control charts designed for monitoring both parameters of a LTPHR process characterized by the baseline survival function $\bar{F}(x)$ and the parameter vector (μ,λ). These charts were developed based on progressively Type-II censored data with a censoring scheme denoted as $r=(r_{1},\ldots ,r_{m})$ . The control charts include: an EWMA chart based on the likelihood ratio statistic (EWMA-LR), an EWMA chart utilizing maximum likelihood estimators (EWMA-Max-MLE), an EWMA chart based on the Manhattan distance (EWMA-MD), and a chart based on a weighted log-likelihood ratio statistic (WL).

To facilitate the implementation of the proposed control charts, we present a Monte Carlo algorithm that allows for the specification of the control chart for a given expected value ${\text{ARL}}_{0}$.

We conducted a comprehensive Monte Carlo simulation study to evaluate the performance of the proposed control charts. This study involved comparing the OOC ARL and the SDRL across various scenarios. The simulation results demonstrate that the WL control chart consistently outperforms the others for monitoring the LTPHR process, regardless of the baseline survival function $\bar{F}(x)$, number of units *n*, and the number of the observed failure times *m*.

Additionally, we applied our methods to analyze the lifetimes of two groups of male RFM strain mice, further illustrating their practical utility. In this example, the WL chart again showed superior performance.

A key advantage of our proposed control charts is their reliance solely on $\bar{F}(x)$, the IC values parameters $\mu_{0}$ and $\lambda_{0}$, the smoothing parameter γ, and the nominal ${\text{ARL}}_{0}$ . Consequently, they do not depend on the specific censoring scheme employed.

The extension of the proposed control charts to self-starting schemes, along with the development of other censoring mechanisms, such as progressive hybrid censoring and adaptive progressive censoring, may be the focus of future researches.

## 6 Appendix

### 6.1 The R code of EWMA-LR chart

####### Computimg EL0

n=5; m=3     # insert n and m

d1=rexp(10000000)

d2=rchisq(rep,2*m-2)/(2*m)

LR=m*(d2-log(d2)-1)+d1

mean(LR)

####### Computimg ARL0 (IC) or ARL1 (OOC)

n=5; m=3           # insert n and m

EL0=2.0271         # insert EL0

h.EWMA.LR=2.6606   # insert the obtained h based on ARL0

delta1=-.2; delta2=.8  # set dalta1=0 and

dalta2=1 (IC) to obtain ARL0

gamma=.05          # insert the smoothing parameter gamma

Fbmu0=.8; lambda0=1 # inser Fbar(mu0) (IC) and lambda0 (IC)

rep=20000          # number of replication

Fbmu1=Fbmu0*exp(-delta1/lambda0)

lambda1=lambda0/delta2

L=c()

for(k in 1:rep){

i=0

EL=EL0

while(EL<h.EWMA.LR){

Fbmu1hat=Fbmu1*exp(-rexp(1,n*lambda1))

lambda1hat=2*m*lambda1/rchisq(1,2*m-2)

d1=n*lambda0*log(Fbmu0/Fbmu1hat)

d2=lambda0/lambda1hat

if(d1>0) LR=m*(d2-log(d2)-1)+d1 else LR=10000

EL=(1-gamma)*EL+gamma*LR

i=i+1

}

L[k]=i

}

mean(L)

sd(L)

### 6.2 The R code of EWMA-Max-MLE chart

#====== Computimg ARL0 (IC) or ARL1 (OOC)

n=5; m=3

EM0=1.128379

h.EWMA.Max.MLE=1.3444

delta1=-.2; delta2=.8

gamma=.05

Fbmu0=.8; lambda0=1

rep=20000

Fbmu1=Fbmu0*exp(-delta1/lambda0)

lambda1=lambda0/delta2

L=c()

for(k in 1:rep){

i=0

EM=EM0

while(EM<h.EWMA.Max.MLE){

Fbmu1hat=Fbmu1*exp(-rexp(1,n*lambda1))

lambda1hat=2*m*lambda1/rchisq(1,2*m-2)

Z1=Fbmu1hat/Fbmu0

Z2=2*m*lambda0/lambda1hat

T1=qnorm(pbeta(Z1,n*lambda0,1))

T2=qnorm(pchisq(Z2,2*m-2))

S=max(abs(T1),abs(T2))

EM=(1-gamma)*EM+gamma*S

i=i+1

}

L[k]=i

}

mean(L)

sd(L)

### 6.3 The R code of EWMA-MD chart

#====== Computimg EMD0

n=5; m=3 # insert n and m

lambda0=1 # insert lambda0

d1=log(rbeta(10000000,n**lambda0,1))

d2=rchisq(10000000,2*m-2)/(2*m)

MD=lambda0*abs(d1)+abs(d2-1)

mean(MD)

#====== Computimg ARL0 (IC) or ARL1 (OOC)

n=5; m=3

EM0=.6992

h.EWMA.MD=.8249

delta1=-.2; delta2=.8

gamma=.05

Fbmu0=.8; lambda0=1

rep=20000

Fbmu1=Fbmu0*exp(-delta1/lambda0)

lambda1=lambda0/delta2

L=c()

for(k in 1:rep){

i=0

EM=EM0

while(EM<h.EWMA.MD){

Fbmu1hat=Fbmu1*exp(-rexp(1,n*lambda1))

lambda1hat=2*m*lambda1/rchisq(1,2*m-2)

S=n^2*lambda0^2*(log(Fbmu0)-log(Fbmu1hat))^2+

m^2/(m-1)*(lambda0/lambda1hat-1)^2-2-m/(m-1)

EM=(1-gamma)*EM+gamma*S

i=i+1

}

L[k]=i

}

mean(L)

sd(L)

### 6.4 The R code of WL chart

#====== Computimg ARL0 (IC) or ARL1 (OOC)

n=5; m=3

h.WL=.1141

delta1=-.2; delta2=.8

gamma=.05

Fbmu0=.8; lambda0=1

rep=20000

Fbmu1=Fbmu0*exp(-delta1/lambda0)

lambda1=lambda0/delta2

L=c()

for(k in 1:rep){

K=matrix(,nr=100000,nc=2)

i=1

repeat{

Fbmu1hat=Fbmu1*exp(-rexp(1,n*lambda1))

lambda1hat=2*m*lambda1/rchisq(1,2*m-2)

K[i,]=c(Fbmu1hat,lambda1hat)

w=gamma*(1-gamma)^((i-1):0)

W=sum(w)

Fbmu1tilda=max(K[1:i,1])

lambda1tilda=m*W/sum(w*(m/K[1:i,2]-n*log(K[1:i,1]/Fbmu1tilda)))

d1=n*lambda0*log(Fbmu0/Fbmu1tilda)

d2=lambda0/lambda1tilda

if(d1>0) WL=W*(m*(d2-log(d2)-1)+d1) else WL=10000

if(WL > h.WL) {AR=i; break} else i=i+1

}

L[k]=AR

}

mean(L)

sd(L)
